# Spike sorting in the presence of stimulation artifacts: a dynamical control systems approach

**DOI:** 10.1088/1741-2552/ad228f

**Published:** 2024-02-09

**Authors:** Mohammad Shokri, Alex R Gogliettino, Paweł Hottowy, Alexander Sher, Alan M Litke, E J Chichilnisky, Sérgio Pequito, Dante Muratore

**Affiliations:** 1 Delft Center for Systems and Control, Delft University of Technology, Delft 2628 CN, The Netherlands; 2 Neurosciences PhD Program, Stanford University, Stanford, CA 94305, United States of America; 3 Hansen Experimental Physics Laboratory, Stanford University, Stanford, CA 94305, United States of America; 4 Faculty of Physics and Applied Computer Science, AGH University of Krakow, Krakow, Poland; 5 Santa Cruz Institute for Particle Physics, University of California, Santa Cruz, CA, United States of America; 6 Departments of Neurosurgery and Ophthalmology, Stanford University, Stanford, CA 94305, United States of America; 7 Division of Systems and Control, Department of Information Technology, Uppsala University, 751 05 Uppsala, Sweden; 8 Microelectronics Department, Delft University of Technology, Delft 2628 CN, The Netherlands

**Keywords:** Bi-directional neural interface, spike sorting, stimulation artifact, dynamical control systems

## Abstract

*Objective*. Bi-directional electronic neural interfaces, capable of both electrical recording and stimulation, communicate with the nervous system to permit precise calibration of electrical inputs by capturing the evoked neural responses. However, one significant challenge is that stimulation artifacts often mask the actual neural signals. To address this issue, we introduce a novel approach that employs dynamical control systems to detect and decipher electrically evoked neural activity despite the presence of electrical artifacts. *Approach*. Our proposed method leverages the unique spatiotemporal patterns of neural activity and electrical artifacts to distinguish and identify individual neural spikes. We designed distinctive dynamical models for both the stimulation artifact and each neuron observed during spontaneous neural activity. We can estimate which neurons were active by analyzing the recorded voltage responses across multiple electrodes post-stimulation. This technique also allows us to exclude signals from electrodes heavily affected by stimulation artifacts, such as the stimulating electrode itself, yet still accurately differentiate between evoked spikes and electrical artifacts. *Main results*. We applied our method to high-density multi-electrode recordings from the primate retina in an *ex vivo* setup, using a grid of 512 electrodes. Through repeated electrical stimulations at varying amplitudes, we were able to construct activation curves for each neuron. The curves obtained with our method closely resembled those derived from manual spike sorting. Additionally, the stimulation thresholds we estimated strongly agreed with those determined through manual analysis, demonstrating high reliability ($R^2 = 0.951$ for human 1 and $R^2 = 0.944$ for human 2). *Significance*. Our method can effectively separate evoked neural spikes from stimulation artifacts by exploiting the distinct spatiotemporal propagation patterns captured by a dense, large-scale multi-electrode array. This technique holds promise for future applications in real-time closed-loop stimulation systems and for managing multi-channel stimulation strategies.

## 1. Introduction

Bi-directional neural interfaces (BNIs) play an increasingly important role in neurotechnology to communicate with the nervous system using multi-electrode arrays (MEAs). These interfaces promise to revolutionize scientific discovery and clinical therapeutics through closed-loop neuromodulation [14, 35, 37, 49, 70]. Specially, a BNI performs two major tasks. On the one hand, it stimulates neurons to produce targeted patterns of neural activity that are useful for the scientific or clinical application [20, 33, 47]. On the other hand, it performs electrical recording to observe natural neural activity and to calibrate the activity evoked by the interface [17, 36, 42, 48, 59, 60, 66, 73].

A critical challenge in recording electrically-evoked activity is that the voltage produced by injecting the current into the electrode–electrolyte impedance produces a stimulation artifact that is often large enough to obscure the evoked neural signal of interest [28]. Because of the large time constants of the electrode-tissue impedance, the artifact can last for several milliseconds after stimulation [23, 45] and can thus overlap in time with evoked spikes. This substantially complicates the process of identifying and segregating spikes from different cells (spike sorting). Therefore, the artifact and the neural activity of interest must be distinguished [50, 56].

Several approaches have been proposed to use the *temporal* properties of spikes and artifacts to perform spike sorting [6, 8, 17, 38, 58, 67, 68]. In template subtraction methods, the estimated artifacts are subtracted from the measurements to isolate neural activity [13, 23, 46, 65] and identify spikes [40]. However, obtaining templates of the artifact in isolation is not always possible [51].

**Figure 1.**
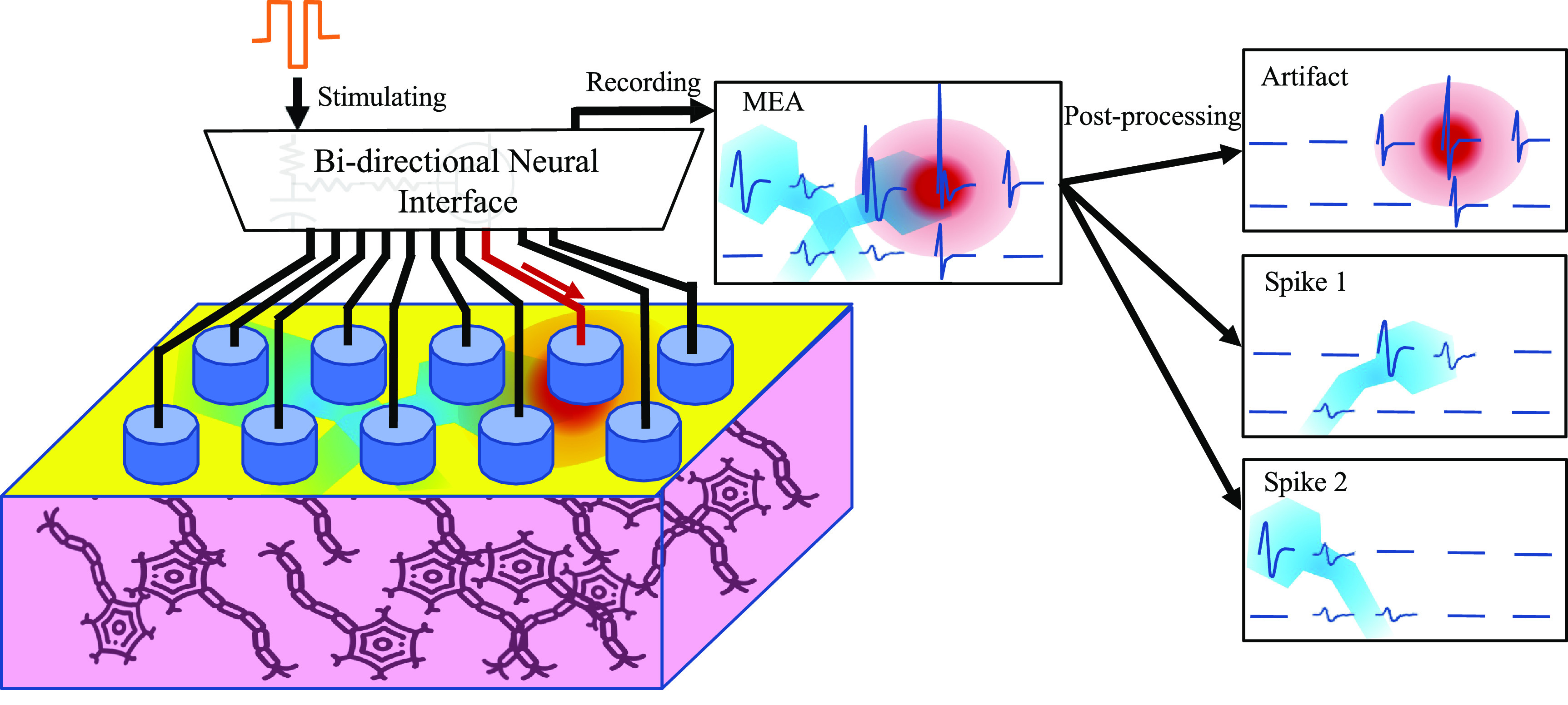
Eye-bird view of spike sorting in the presence of stimulation artifacts. A charge-balanced tri-phasic waveform (indicated in red) is used by the stimulator in this paper. The neural interface records the voltage immediately after the stimulation ends. The recordings include the residual artifact(s), and evoked and/or spontaneous spikes (MEA regions of interest are shown in red and blue, respectively). Post-processing is needed to analyze the recordings and separate the effect of different phenomena (spikes and artifacts).

However, relatively little has been done to exploit the distinct *spatiotemporal* propagation of electrical artifacts and spikes [45, 54]. Here, we propose a novel approach using dynamical control systems to model the spatiotemporal propagation of spikes and artifacts and exploit their differences to identify evoked neural activity as shown in figure 1. Specifically, we design a unique dynamical model for the stimulation artifact and for each neuron recorded during spontaneous activity. Then, to identify evoked spikes after stimulation, we estimate which combination of dynamical models (i.e. which neurons firing) were most likely to produce the recorded voltage response across all electrodes. Notably, the method does not require recordings from the stimulation electrode itself, which typically has an artifact that saturates recording electronics, enabling lower-power electronics. We demonstrate the effectiveness of the proposed approach on large-scale multi-electrode *ex vivo* recordings from primate retina, and compare the results to human-supervised spike sorting.

## 2. Materials and methods

### 2.1. Experimental setup and data description

We analyzed voltage recordings from primate retinal ganglion cells (RGCs) during epiretinal electrical stimulation, from a single retinal preparation. Eyes were obtained from terminally anesthetized macaque monkeys (*Macaca mulatta, Macaca fascicularis*) used by other researchers, in accordance with Institutional Animal Care and Use Committee guidelines. Further details on the experimental preparation have been described previously [21, 22, 44].

**Figure 2.**
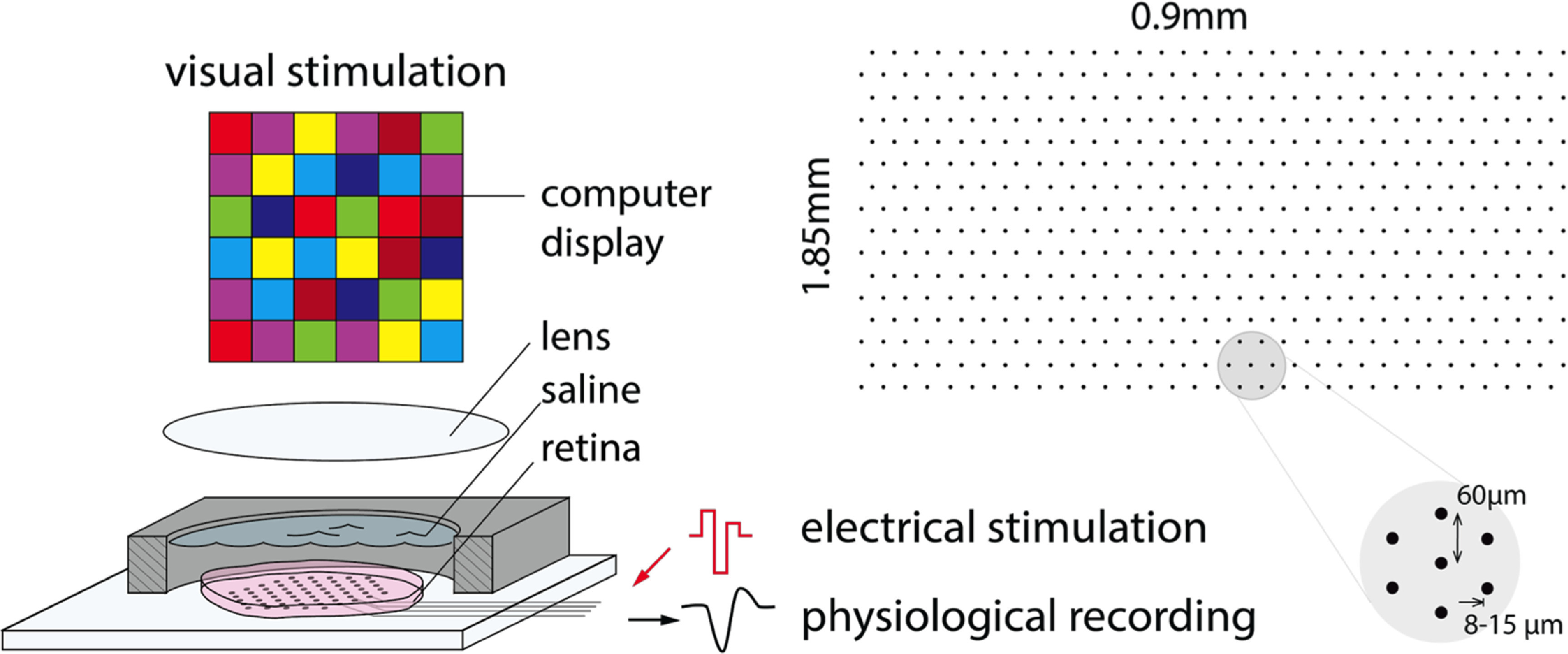
Experimental setting illustration. The retina was isolated from the retinal pigment epithelium and placed RGC side down on a custom high-density multi-electrode array system with 512 electrodes (60 *µ*m pitch, 8–15 *µ*m diameter). The retina was then stimulated with a white noise visual stimulus using a computer display and lenses while recording on each electrode simultaneously.

**Figure 3.**
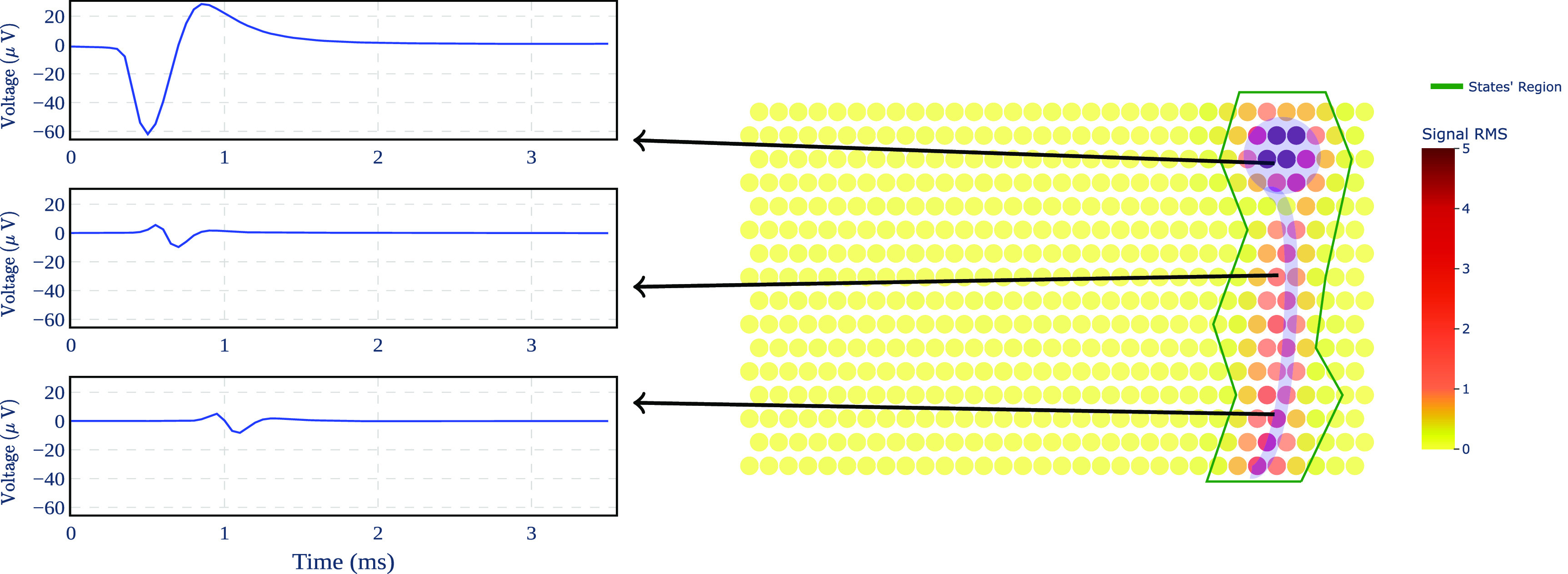
EI data for neuron 18: (left) time-series for three different electrodes, (right) MEA heatmap with the neuron depicted in shaded blue and green contours for the relevant electrodes. The heatmap shows the footprint of the neuron on the MEA; the color encodes the root mean square (RMS) of each electrode’s recordings when a spike from the putative neuron is present.

The retina was isolated from the retinal pigment epithelium and placed RGC side down on a custom high-density MEA system with 512 electrodes (60 *µ*m pitch, 8–15 *µ*m diameter) [28, 42]. The retina was then stimulated with a white noise visual stimulus using a computer display and lenses while recording on each electrode simultaneously—see figure 2. Raw signals were amplified, filtered (43–5000 Hz), multiplexed, digitized (20 kHz) and stored for offline analysis. A custom spike sorting procedure [42] was applied to the recordings to identify and segregate spikes from individual RGCs. The spike-triggered average (STA) stimulus was then computed for each RGC to classify functionally-distinct cell types [10, 11, 21, 61]. For each cell, the electrical image (EI), or the average spatiotemporal pattern of activity associated with a cell’s spike, was computed by averaging the voltage traces during the time of each cell’s spike [42, 57]. EIs for 25 distinct neurons recorded over 71 samples (3.55 ms) were examined (figure 3).

To characterize RGC responses to epiretinal electrical stimulation, the retina was electrically stimulated by injecting a brief current pulse (charge balanced, positive first, triphasic, 50 *µ*s per phase in relative ratios of 2:-3:1) through one electrode at a time in a random sequence while recording on all electrodes simultaneously [21, 22, 33, 44]. Thus, 39 current amplitudes (0.1–4.1 *µ*A on the second phase, log spacing) were applied, with each amplitude repeated 25 times.

**Figure 4.**
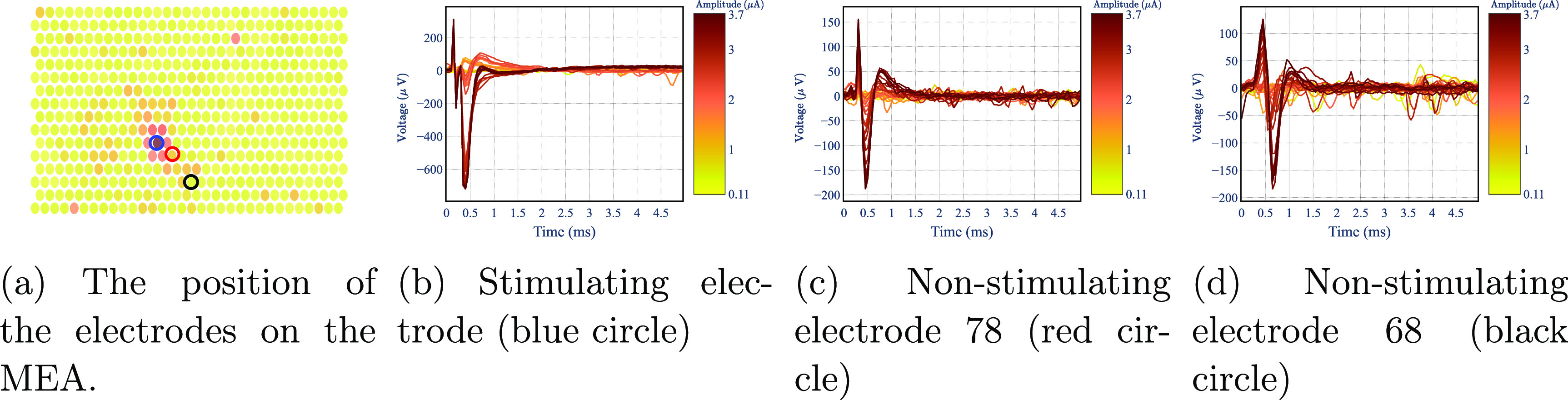
Recordings of a stimulating electrode and two non-stimulating electrodes for different stimuli amplitudes (color-coded).

The electrical artifact associated with electrical stimulation (figure 4) precludes the use of standard spike sorting techniques because it is temporally correlated with and occupies a similar frequency band as neuronal spikes [45]. Furthermore, particularly on the stimulating electrode, the duration of the artifact is nearly 2 ms (figure 4(a)), exceeding the typical response latency of RGCs to electrical stimulation (0.4–0.6 ms) [45], complicating further the analysis of responses. To establish a human-curated set of labeled responses to electrical stimulation against which to compare the algorithm developed here, a semi-automated procedure was performed by two human observers, as described previously [33]. First, recorded traces after stimulus onset (55 samples or 2.75 ms) were considered for analysis. For each cell-electrode pair, clustering was performed on the traces from the trials at a single current level. The trials were grouped into two clusters: one that elicited spikes and another that resembled artifact only. An estimate of the artifact was then calculated from this second cluster and subtracted from trials containing putative spikes. This procedure was repeated for each current amplitude. Then, the artifact-subtracted traces, along with template waveforms obtained from the EI, were visually inspected by each human observer to determine whether the artifact-subtracted data resembled the template of the cell of interest (i.e. an electrically-elicited spike) or not (i.e. no response). Each human observer analyzed a set of 10 cell-electrode pairs from five different neurons.

### 2.2. Dynamical systems approach

We use dynamical systems to model the spatiotemporal evolution of the voltage recorded by the MEA in the presence of spikes from one or more neurons and in the presence of a stimulation artifact [30, 32, 52].

#### 2.2.1. EI models

The EI *model* for a given neuron *n* is a set of equations (e.g. equation (1)) that describes the propagation of the voltage across all *E* = 512 electrodes when the neuron fires a spike. The model consists of a predefined *input* vector $u_n^t \in \mathbb{R}^2$ that initiates the dynamical system, the *state* of the neuron $x_n^t\in \mathbb{R}^{E_n}$ at each point in time defined over a subset of the electrodes relevant for the cell ($E_n\lt E$, see green region in figure 3), and two matrices *A_n_* and *B_n_* that describe the evolution of $x_n^t$ over time. Finally, the *output* $y_n^t \in \mathbb{R}^E$ indicates the voltage contribution of neuron *n* to all electrodes on the array (the output is set to zero for the electrodes that are not relevant for the cell). Thus, the dynamical model of the EI can be described as

\begin{align*} \begin{aligned} x_n^{t+1} &amp; = A_n x_n^t + B_n u_n^t, \\ y_n^t &amp; = C_n x_n^t, \end{aligned} \end{align*}

where the matrix *A_n_* defines how the state of the neuron at the next time step depends on the current state, and captures the spatiotemporal correlation between the electrodes. The matrix *B_n_* defines how the next state depends on the input, and *C_n_* defines how the output depends on the current state.

The model parameters are learned such that if the input templates $r_n^t$ are injected, the model generates outputs (i.e. the voltage on each electrode) that approximately match the EI of neuron *n*. The input templates are two unit-magnitude pulses that trigger rising and falling phases of the spike, respectively—see appendix A for details on modeling and parameter learning.

**Figure 5.**
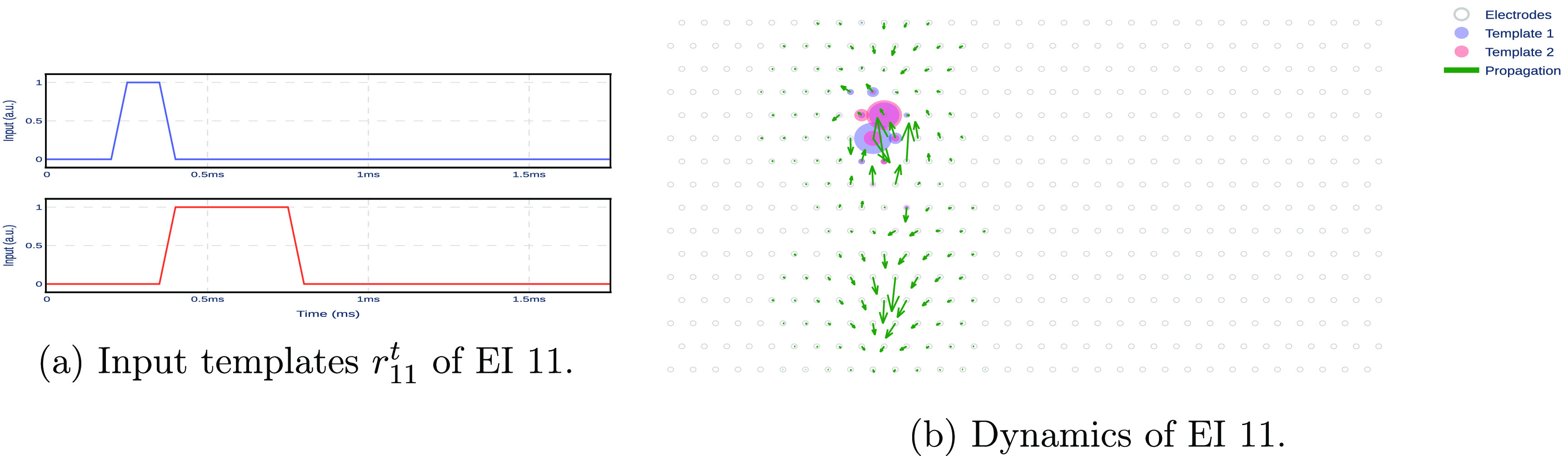
Input templates and their effect on the EI model. (a) The input template includes two pulses. (b) The red and blue circles show the effect of the input templates on the electrodes, as defined by matrix *B_n_*. The arrows show the spatial propagation of the model across electrodes, as defined by matrix *A_n_*.

The input templates were determined based on the dynamics of extracellular action potentials. Specifically, we observed that the recorded spike exhibits distinct upward and downward patterns due to the opening and closing of potassium and sodium channels in the cell’s membrane. To capture these dynamics, we used two heuristic-based templates, drawing upon expected input-output responses of linear systems that align with the shapes of the recorded spike—see figure 5.

#### 2.2.2. Artifact model

**Figure B5.**
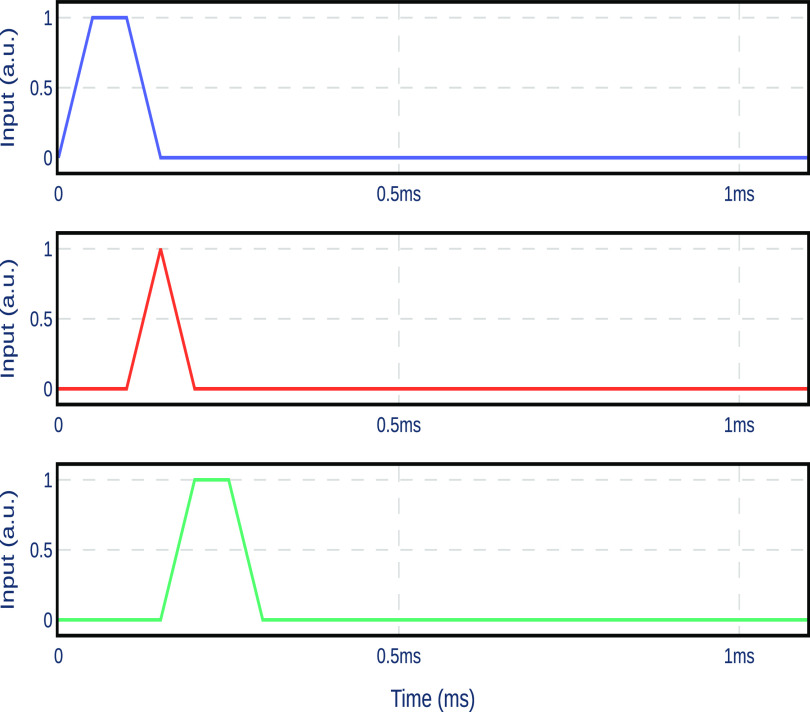
Input templates $r_a^t$ of artifact. The blue, red, and green graphs are the first, second, and third entries of the vector $r_a^t$, respectively.

The artifact *model* describes the propagation of voltage across the electrodes caused by artifacts due to electrical stimulation. Similarly to the EI model, the output $y_a^t \in \mathbb{R}^E$ indicates the voltage recorded on all electrodes immediately after stimulation. The empirically observed artifact propagates radially outward from the stimulating electrode and decays over space. Hence, only the closest electrodes $E_a\lt E$ to the stimulating electrode will record the artifact and are considered as the states of the model, namely $x_a^t\in \mathbb{R}^{E_a}$ at time *t*. Moreover, the stimulating electrode shows discontinuities with respect to the stimulus amplitude due to the design of the stimulation system (see figure 4(b)). Thus, we discard the stimulating electrode from the state variables. As with the EI model, $y_a^t$ is equal to state variable $x_a^t$ for electrodes *E_a_*, and zero for the other electrodes. As with the EI model, the artifact model is learned such that an approximation to the artifact is observed in the output *y_a_* if the input $u_a^t$ has the template $r_a^t \in \mathbb{R}^3$ (figure B5) linearly scaled by the stimulus amplitude *q*. Hence, for the stimulus amplitude *q*, the input to the model is $u_a^t = q r_a^t$. In summary, the dynamical model of the artifact is given by

**Figure 6.**
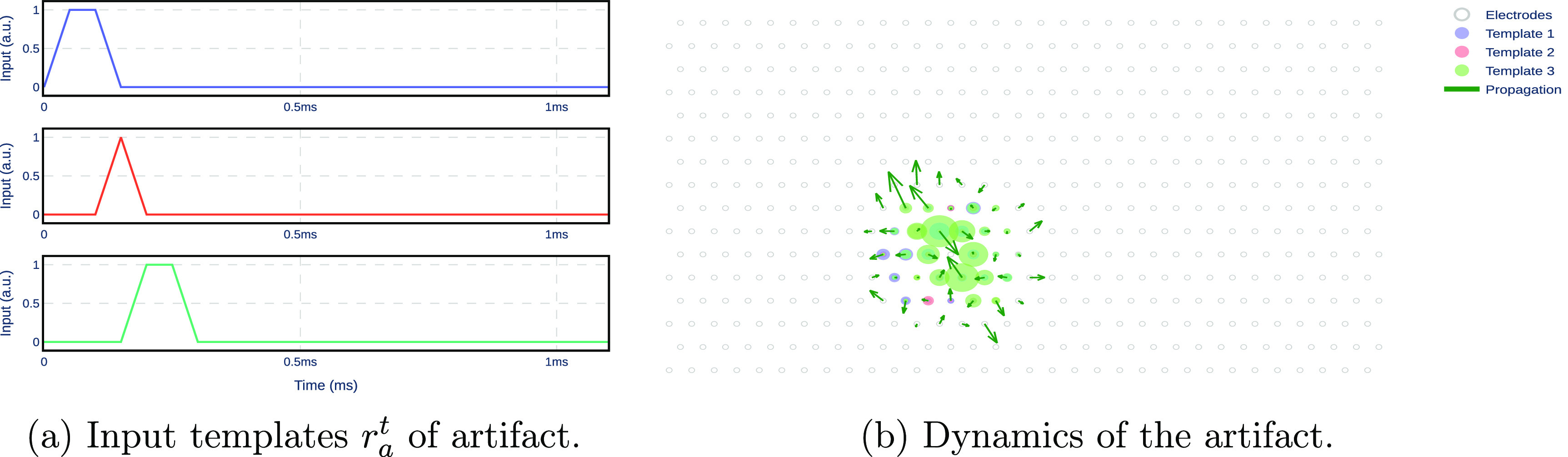
Input templates and their effect on the artifact model. (a) The input template includes three pulses. (b) The red and blue circles show the effect of the template inputs on the electrodes, as defined by matrix *B_n_*. The arrows show the spatial propagation of the model across electrodes, as defined by matrix *A_n_*.

\begin{align*} \begin{aligned} x_a^{t+1} &amp; = A_a x_a^t + B_a u_a^t, \\ y_a^t &amp; = C_a x_a^t, \end{aligned} \end{align*}

where the vector $u_a^t \in \mathbb{R}^3$ is the input of the model, the *B_a_* matrix defines how the next state depends on the input of the model, the matrix *A_a_* defines how the next state depends on the current state, and the matrix *C_a_* defines how the output depends on the current state. *A_a_*, *B_a_* and *C_a_* are obtained from the average of stimulation data across—see appendix B for details on modeling and parameter learning. Figure 6(b) demonstrates how the artifact model characterizes the propagation of the artifact on MEAs.

For designing the input templates, we fitted the recorded steady-state response during stimulation in our experimental setup. This resulted in a template with three inputs specific to our experimental setup. A similar curve-fitting exercise should be repeated for a different experimental setup, which might result in different input templates.

#### 2.2.3. Aggregate model

**Figure 7.**
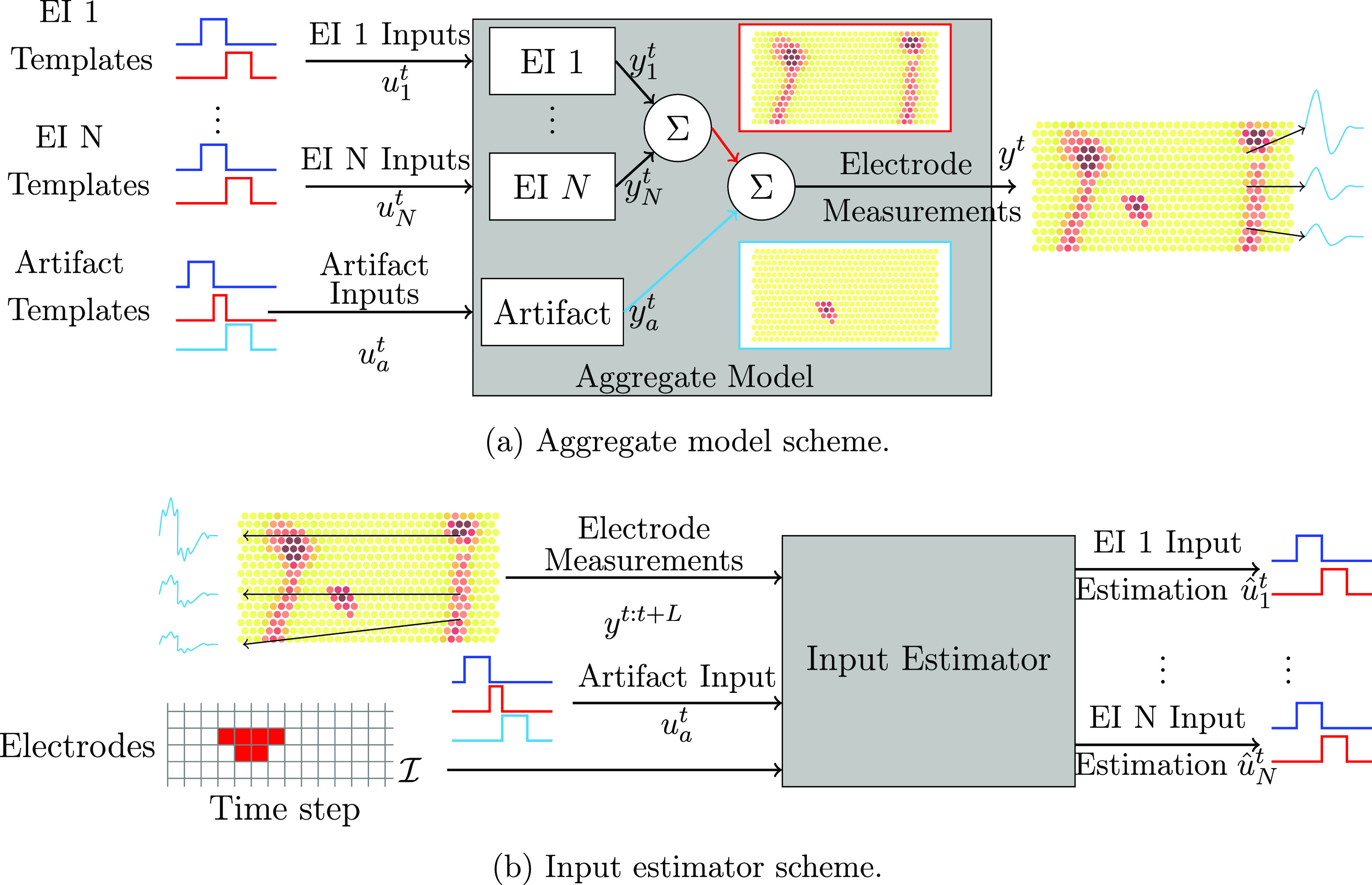
Overview of the proposed spike sorting using dynamical systems and input estimator. (a) We assume that the measured output is the superposition of the output of dynamical models for different EIs and artifacts as driven by predefined input templates. The combination of the EI models and artifact models is the aggregate model. (b) We propose a method to estimate the set of input templates that led to a specific measurement (see details in section 2.2.4). The input estimator requires the electrode measurements immediately after stimulation, the aggregate model, and the list of corrupted electrodes to ignore (typically, the stimulating and nearby electrodes shown in red in the grid). Since we know *a priori* that stimulation happened, the artifact template is automatically estimated. By inferring which inputs are active (i.e. they are estimated to be equal to the input template), we can identify which neuron(s) are firing; hence, we can perform spike sorting.

The aggregate model describes the propagation of the voltage across electrodes in the presence of both stimulation artifact and neurons firing. This model is a linear combination of the EI model for all neurons, the artifact model and the measurement noise (superposition assumption in MEA recordings [58]). As shown in figure 7(a), the aggregate model outputs the electrode measurements for multiple input templates. Specifically, the observed outputs on the electrodes are caused by injecting templates of the different sub-models (EI and/or artifact models) in the aggregate model. Thus, the aggregate model is given by

\begin{align*} \begin{aligned} {\tilde x}^{t+1} &amp; = {\tilde A} {\tilde x}^t + {\tilde B} {\tilde u}^t + {\tilde B}^{\prime} u_a^t, \\ y^t &amp; = {\tilde C} {\tilde x}^t + w^t, \end{aligned} \end{align*}

where ${\tilde x} = \left[ x_1^\top,\dots, x_N^\top, x_a^\top \right]^\top$ is the aggregate state, ${\tilde u} = \left[ u_1^\top,\dots, u_N^\top \right]^\top$ is the aggregate input of the EI models, $u_a^t$ is the input of the artifact model, and $w^t\in\mathbb{R}^E$ denotes the measurement noise. $[{\tilde A},{\tilde B},{\tilde B}^{\prime},{\tilde C}]$ are defined such that the aggregate output *y^t^* satisfies $y^t = \sum_{n = 1}^{N}y_n^t + y_a^t + w^t $—see appendix C for calculation of the matrices.

#### 2.2.4. Input estimator

The aggregate model described in section 2.2.3 determines how the electrodes’ voltage is generated based on the input sequence (figure 7(a)). Conversely, the input estimator leverages the aggregate model to infer (or, equivalently, estimate) the input sequence that likely generated the observed data (figure 7(b)). Notably, the estimated input sequence can be compared to the EI templates to perform spike detection and sorting—i.e. if $u_n = r_n$, then the neuron *n* is firing a spike.

The input estimator utilizes the output data $y^{t:t+L} = \left[ (y^{t})^\top,\dots,(y^{t+L})^\top \right]^\top$ within a forward window *L* to estimate ${\hat u}^t$ of ${\tilde u}^t$, and ${\hat x}^t$ of ${\tilde x}^t$. The estimated EI inputs has the structure ${\hat u}^t = \left[ ({\hat u}_1^t)^\top,\dots,({\hat u}_N^t)^\top \right]^\top$ where ${\hat u}_n^t$ denotes estimated inputs corresponding to EI *n*. Also, it uses the artifact inputs $u_a^t$ as known inputs since the timing of the stimulation is known. Additionally, ${\cal I}$ indicates the indices for the corrupted electrodes that we desire to exclude from the input estimator (i.e. the stimulating electrode in our case). Therefore, the input estimation procedure can be described as

\begin{align*} \begin{aligned} \left({\hat u}^t,{\hat x}^t\right) = f\left(y^{t:t+L},{\hat x}^{t-1}, u_a^t, {\cal I}\right), \end{aligned} \end{align*}

where the function $f(y^{t:t+L},{\hat x}^{t-1}, u_a^t, {\cal I})$ is the so-called input estimator—see appendix D for how the input estimator is designed.

**Figure 8.**
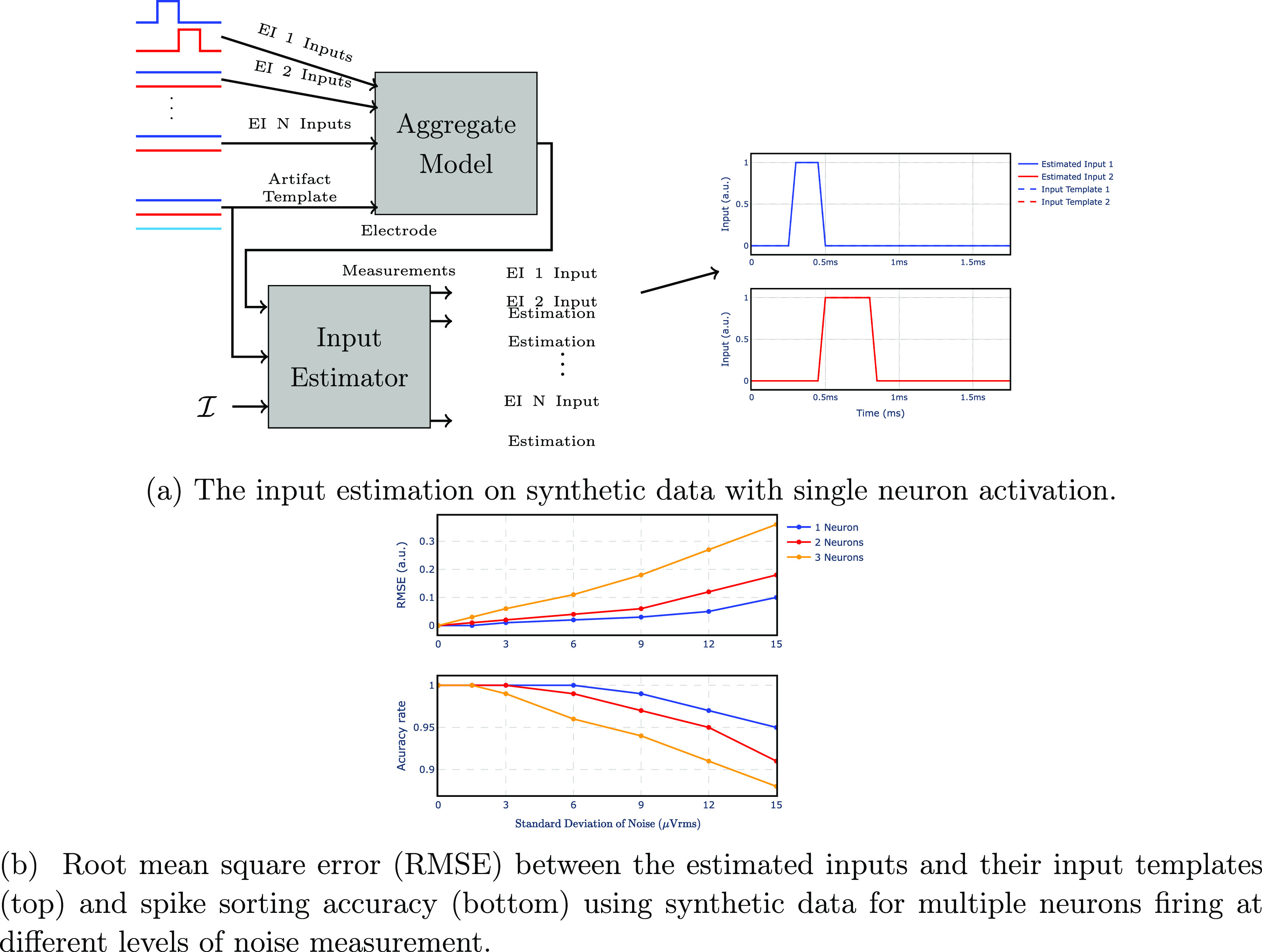
(a) Performance of the input estimator when analyzing synthetic data. The input templates of EI 1 are injected into the aggregate model to generate the synthetic data. The input estimator retrieves inputs that completely overlap with the input templates used to generate the synthetic data (shown on the right). (b) Root mean square error (RMSE) between the input templates and their estimations (top) and spike sorting accuracy (bottom) when one, two, and three neurons fire simultaneously. The simulation uses synthetic data generated by injecting input templates for one, two, and three neurons (out of 25 total neurons) into the aggregate model. The synthetic data is fed to the input estimator, and the RMSE shows how much the estimated inputs deviate from their templates. The simulation is repeated for different measurement noise levels (normal noise with various standard deviations added to synthetic data). As expected, the input estimator does not introduce errors in the noiseless case, even for multiple neurons firing (similar to (a)). As the measurement noise increases, the RMSE increases. Besides, the results suggest that the input retrieval for multiple neurons is more vulnerable to noise than the single-neuron case.

To illustrate and validate the performance of the input estimator for spike sorting, we first consider an example using synthetic data for a single neuron firing, generated by feeding the template for the EI model of one cell to the aggregate model. The generated data is then passed to the input estimator to retrieve which neuron(s) fired. As expected, the estimated input template completely overlaps with the input template of the single EI model used to generate the synthetic data (figure 8(a)). Hence, the input estimator can infer from the synthetic data that the neuron was firing a spike. Section 3.3 validates the input estimator with real noisy data and section 3.4 validates the spike sorting method based on the input estimator with real data contaminated by the stimulation artifact.

The proposed methodology can also detect neural activation while multiple neurons are firing. Figure 8(b) shows the performance of the input estimator on synthetic data. Similar to the results in figure 8(a), the input estimator retrieves the inputs without error for both single and multiple neurons’ activation. To illustrate the sensitivity of input estimation to the electrodes’ noise, we added normal noise to the synthetic data with different standard deviations. As presented in figure 8(b), it is possible to detect three neurons simultaneously despite the fact that higher levels of noise are expected in comparison with single-neuron detection. However, the error also depends on the arrangement of neurons. In particular, the further apart the neurons are, the easier their activation can be detected under possible stimulation artifacts. To perform spike sorting, the input sequence estimated by the input estimator is compared to the input template for each neuron using a similarity function. If its value exceeds a predefined threshold, then we infer that a spike occurred for that specific neuron—see details in appendix G. Figure 8(b) shows that the proposed method is robust enough to perform spike sorting when multiple neurons fire simultaneously.

## 3. Results

This section shows the performance of EI and artifact models, and the input estimator. First, we provide evidence that the EI models are suitable for representing real data accurately. Next, we show the performance of the artifact model for different stimulus amplitudes. We then provide evidence of the input estimator’s ability to predict the correct input sequence with spontaneous activity (i.e. without stimulation artifact) and evoked activity (i.e. with stimulation artifact). Finally, we demonstrate the effectiveness of the proposed spike sorting for different stimulation amplitudes and compare the results to human-supervised spike sorting.

### 3.1. EI models

**Figure 9.**
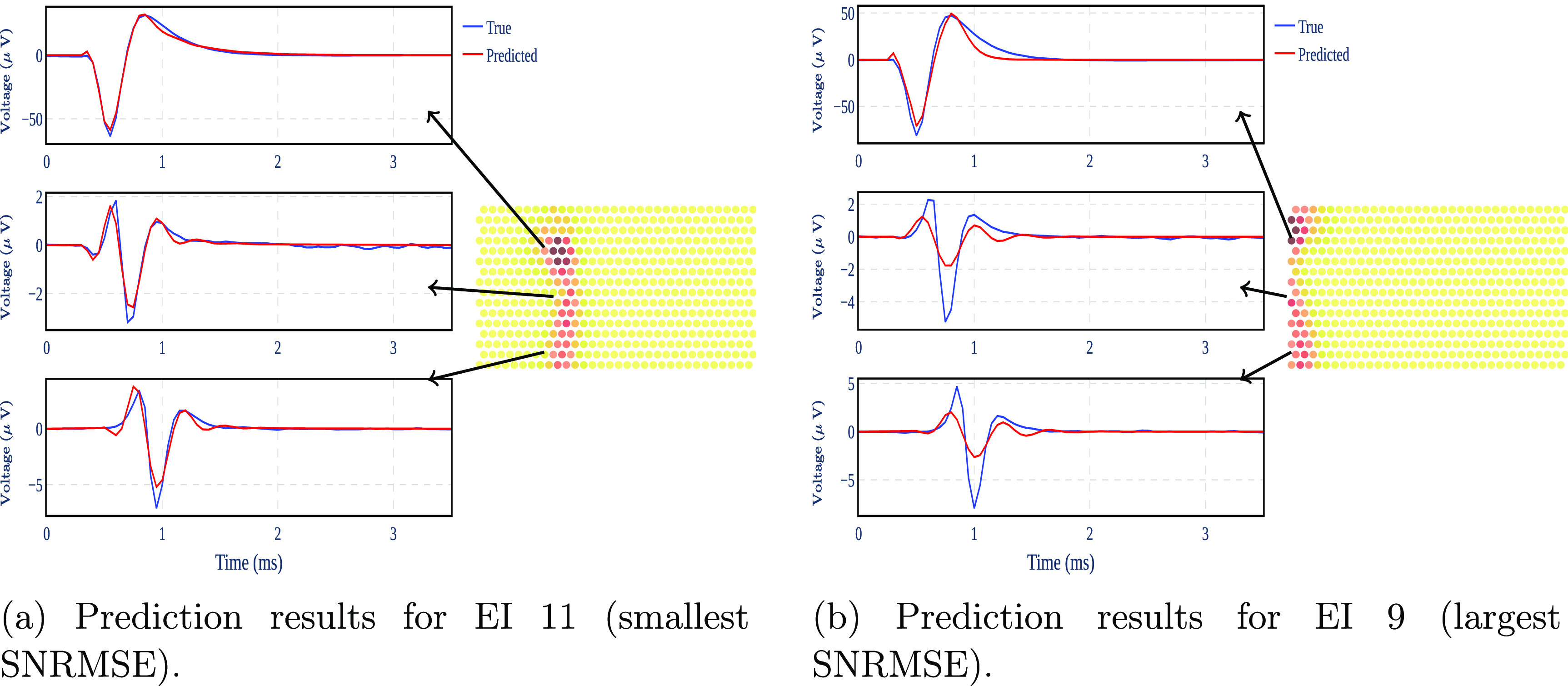
Prediction results from the best and worst performing EI models for three different electrodes. The spatial map of the EI is shown on the MEA heatmap.

To analyze the performance of the EI models, we compare the output of the EI model (i.e. the predicted voltage for all electrodes) with the real measurements of the EI for all neurons. To quantify the quality of the proposed EI models, we use spike normalized root mean square error (SNRMSE): the root mean square of the difference between the real output and estimated output divided by the root mean square of the EI signal—see appendix E. Figure 9 shows the prediction results for the neurons with the lowest (9(b)) and the highest (9(a)) SNRMSE.

**Figure 10.**
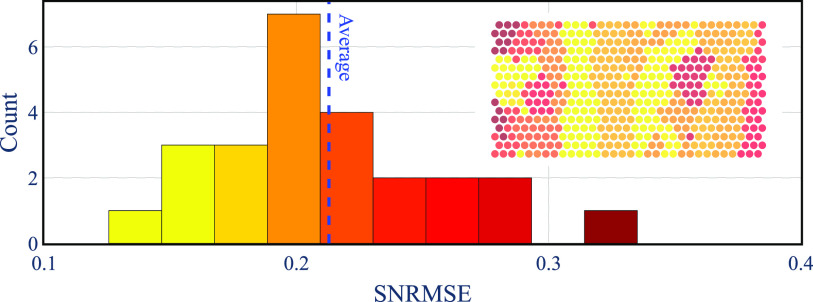
SNRMSE distribution for all EI models. The MEA heatmap shows the neurons’ location based on their SNRMSE. The average SNRMSE is depicted with a blue dashed line.

The generated output from the EI models follows the EI original data for the different electrodes. Notice that the electrode near the soma shows a lower SNRMSE than the other electrodes along the axon. The higher error for the neuron in figure 9(b) may be caused by the location of the corresponding neuron at the edge of the MEA where there are fewer electrodes to capture its dynamical evolution. The right-skewed SNRMSE distribution across all neurons (figure 10) provides evidence that most EI models incur SNRMSE less than the average.

### 3.2. Artifact model

**Figure 11.**
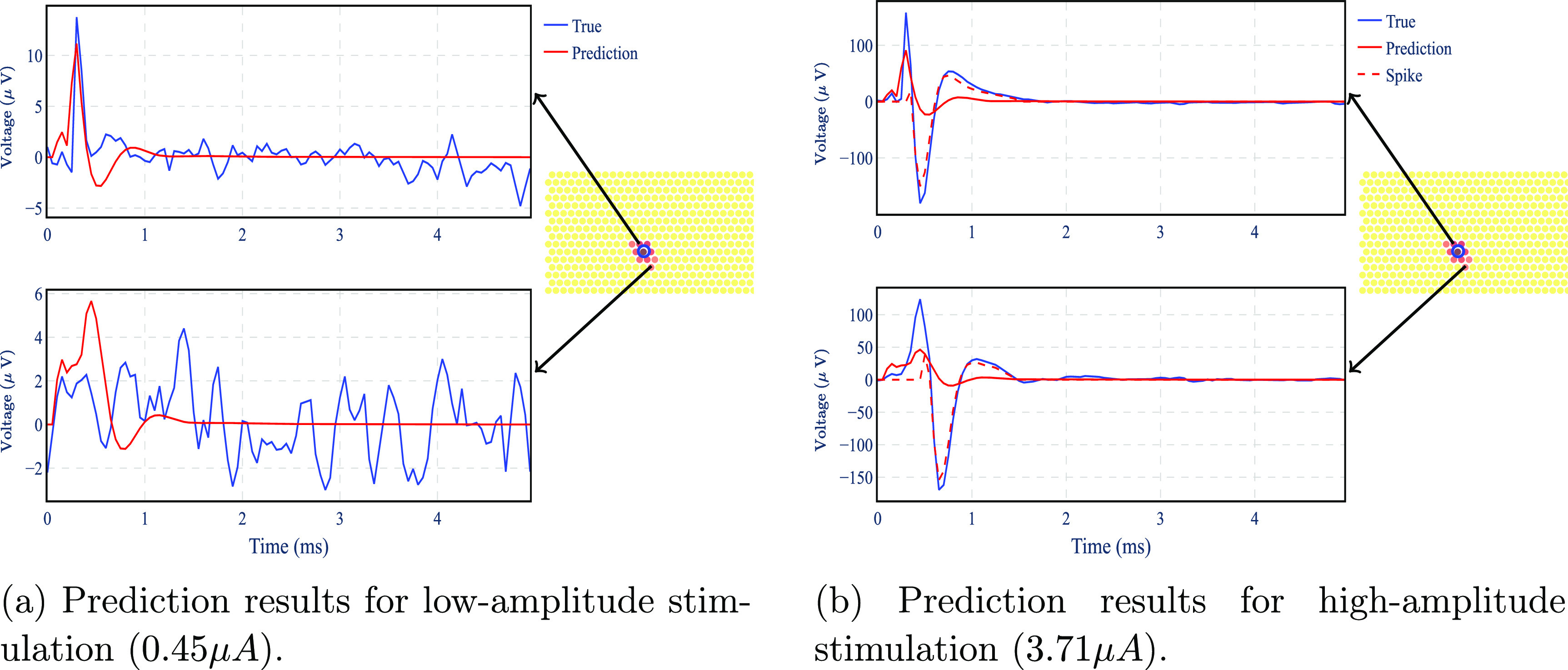
Prediction results of the artifact model for two electrodes at low and high amplitudes. The blue circle indicates the position of the stimulating electrode. The model output is compared with the average stimulation data across all trials. For the high amplitude cases, a putative elicited spike is shown in a red dashed line.

To analyze the performance of the artifact model, we compare the model output to the real measurements for the electrodes close to the stimulating electrode (except for the stimulating electrode itself, which is discarded from our model) for different stimulus amplitudes. Figure 11 shows how the output generated by the simulated model follows the real measurement for two electrodes. At low stimulation amplitude (figure 11(a)), the model follows the average stimulation data for the electrodes. At high stimulation amplitude (figure 11(b)), the model exhibits higher error in a specific interval, possibly due to the presence of an evoked spike superimposed in the recording. This possibility is consistent with the fact that higher stimulation amplitude usually leads to a higher probability of neuron activation.

**Figure 12.**
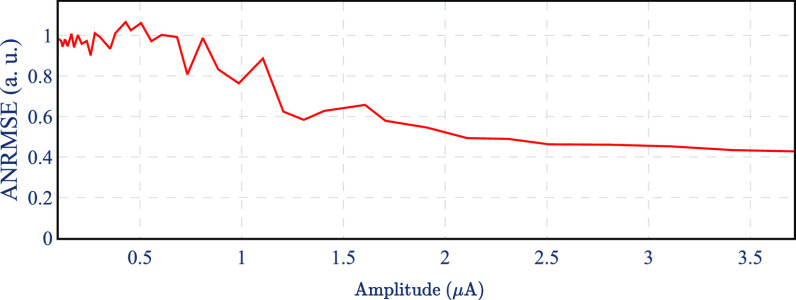
ANRMSE of the artifact model as a function of the stimulation amplitude.

To quantify the performance of the model, we used artifact normalized root mean square error (ANRMSE): the root mean square of the difference between the real output and estimated output divided by the root mean square of the artifact signal—see appendix F. Figure 12 shows the ANRMSE as a function of the stimulation amplitude. The ANRMSE is highest for low stimulation amplitudes, when the artifact is small, and the output is dominated by noise figure 12. However, ANRMSE is reduced for higher stimulation amplitudes since the artifact becomes the dominant contributor to the output, showing that the model is capable of predicting the artifact shape.

### 3.3. Input estimator

**Figure 13.**
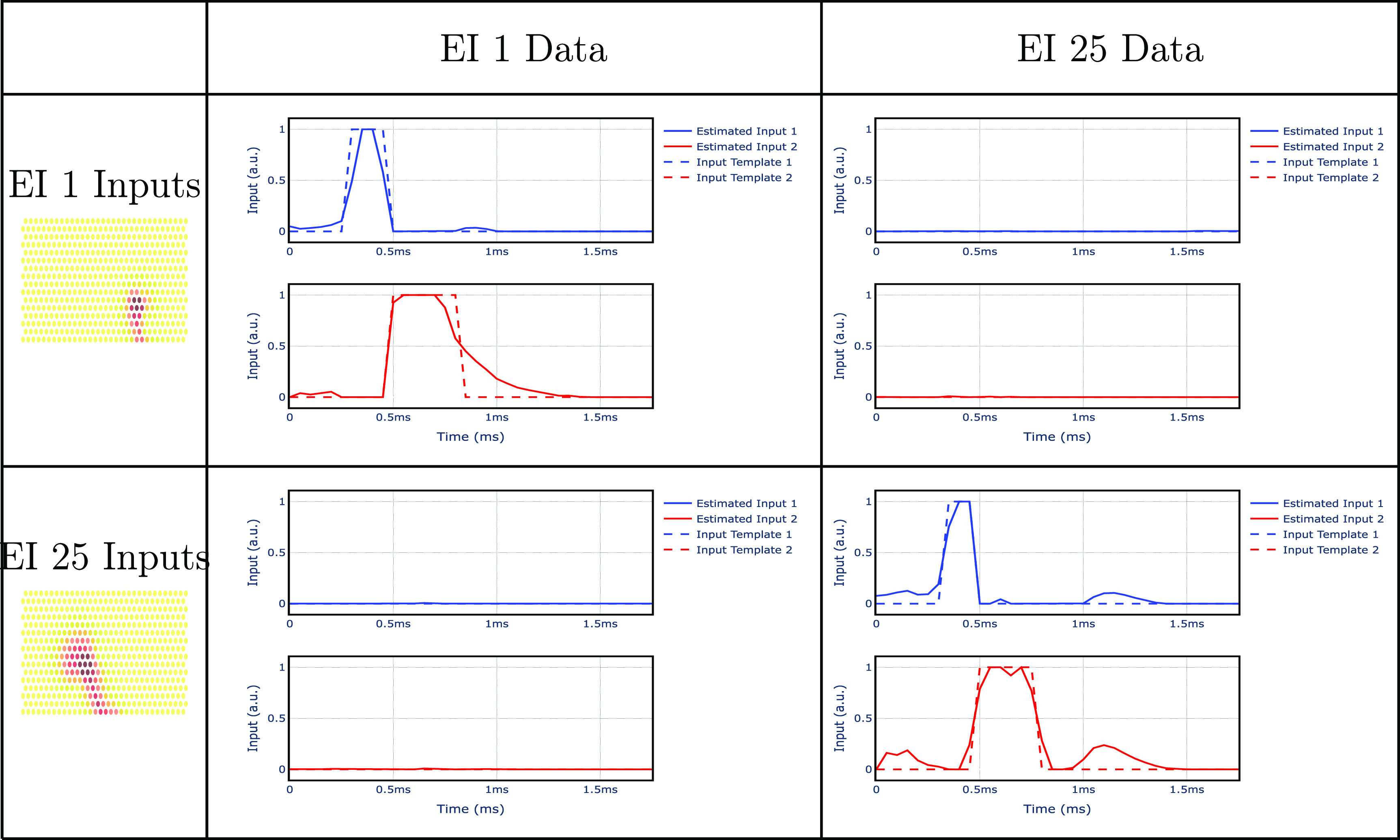
Comparison between retrieved inputs and input templates when ex vivo recordings from EI 1 and EI 25 are fed to the input estimator.

The input estimator can retrieve the input sequences based on the electrodes’ measurements. By comparing the input sequence to the input templates for each neuron, we can retrieve which neuron fired a spike, i.e. perform spike sorting. In section 2.2.4, we showed that the input estimator can retrieve the correct input sequence from synthetic data generated by the aggregate model (figure 8). Here, we show that the input estimator can still recover the correct input sequence from real EI data (figure 13). Notice that this analysis is for measurements without a stimulation artifact. In particular, when analyzing data from EI 1 (i.e. data where only neuron 1 is firing a spike), the input estimator retrieves the input for neuron 1 with a similar shape to its predefined template, suggesting that neuron 1 is firing a spike. In contrast, the estimated input for EI 25 is zero, indicating that neuron 25 is not firing a spike. Additionally, when analyzing data from EI 25 (i.e. data where only neuron 25 is firing a spike), the input estimator retrieves only the input template from neuron 25 and zero from neuron 1.

**Figure 14.**
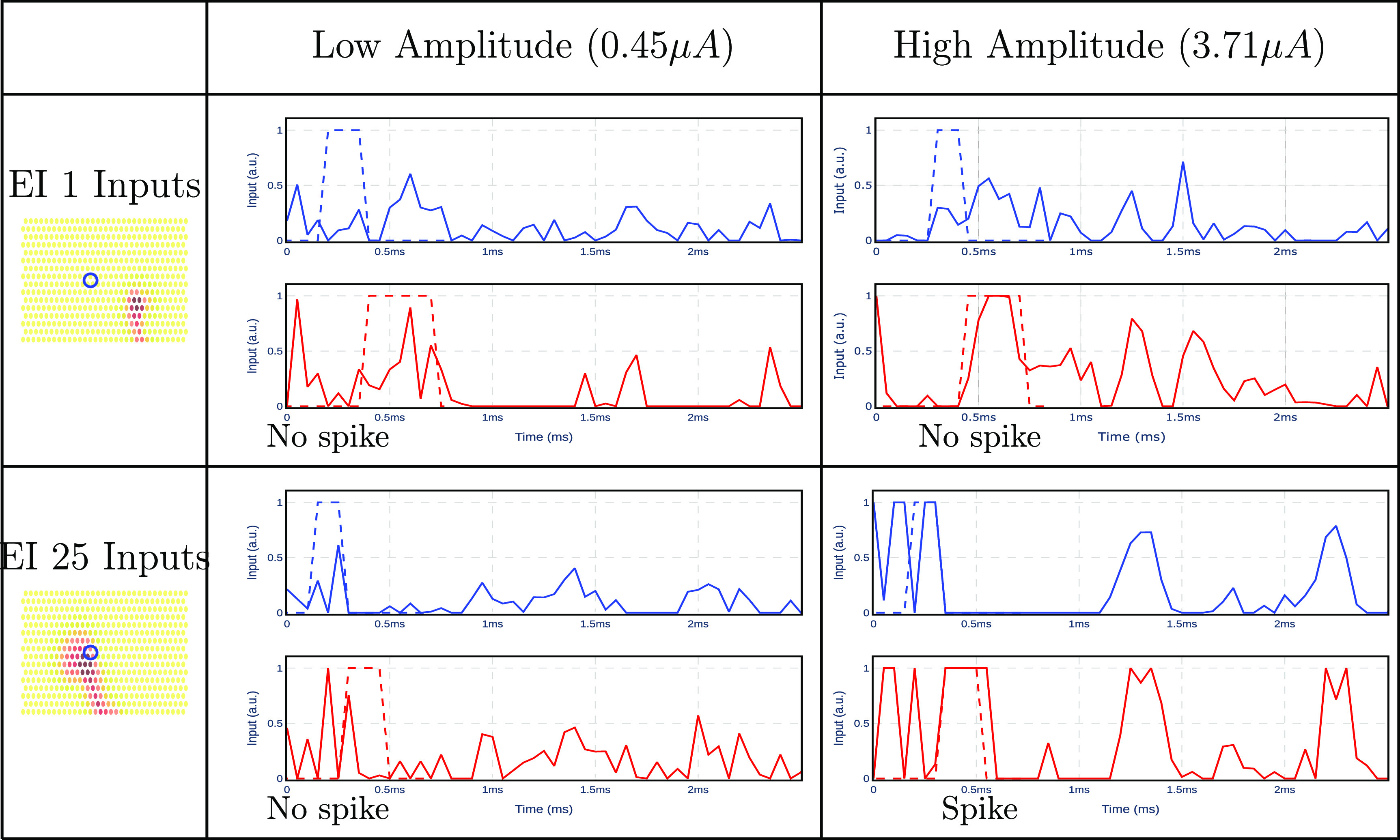
Retrieved inputs of neuron 25 and neuron 1 when ${ex\,\,vivo}$ stimulation data is fed to the input estimator at low amplitude ($0.45~\mu$A) and high amplitude ($3.71~\mu$A). Input templates for each neuron are shown in dashed lines. Neuron 25 is located behind the stimulating electrode (indicated by a circle), and neuron 1 is further away. Using a similarity function, a spike is detected only for neuron 25 at high stimulation amplitude (bottom-right panel).

Now, let us consider measurements immediately after stimulation that contain a stimulation artifact and can contain one or more spikes (figure 14). In this example, the stimulation electrode is near neuron 25 and farther from neuron 1. Hence, it is expected that electrical stimulation is more likely to elicit a spike in neuron 25 than in neuron 1. At low stimulation amplitude, neither neuron fires a spike. At high stimulation amplitudes, however, the retrieved input sequence for neuron 25 suggests that it fired a spike, while still no spike was retrieved for neuron 1.

### 3.4. Spike sorting with stimulation artifact

Here, we perform spike sorting as described in section 2.2.4—see further details in appendix G. By extending this procedure to all stimulation amplitudes over multiple trials, we obtain the activation curve for each neuron–electrode pair: the probability of a neuron firing as a function of the stimulus amplitude applied on a given electrode.

**Figure 15.**
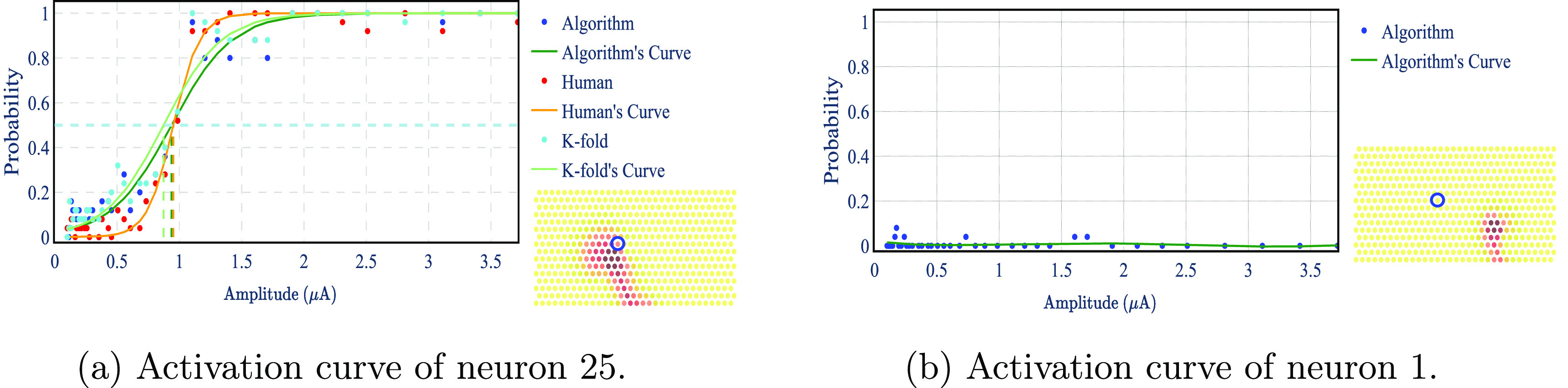
Probability of activation as a function of the stimulation amplitude for neuron 25 and neuron 1. The stimulation threshold (shown with a vertical dashed line) is defined as the amplitude for which the probability of activation is 50% using a sigmoid fit of the scatter plot. The spatial map of the neurons is shown in the MEA, and a blue circle indicates the position of the stimulating electrode. Results are based on the $ex\,\,vivo$ dataset described in section 2.1.

Figure 15 shows an example of the activation curve of neurons 25 and 1 when stimulating using an electrode close to neuron 25 for both the proposed and human spike sorting. The proposed method results in a similar activation threshold to the human result for neuron 25 (defined as the amplitude for which the spiking probability is $50\%$) and no activation for neuron 1. The threshold is extracted by fitting a sigmoid curve to the scatter plot. Although the proposed method finds approximately the same threshold as human spike sorting, it presents certain differences in the sigmoid fit: (1) non-zero probability for low amplitudes, (2) higher variability, and (3) shallower curvature.

As explained in section 3.2, the artifact model is based on the average of all the trials in the stimulation data. To confirm that this is not a problem in the validation of the algorithm, we performed a *K*-fold analysis (*K* = 5) to validate the algorithm using data unseen during training of the model (see *K*-fold curve in figure 15(a)).

**Figure 16.**
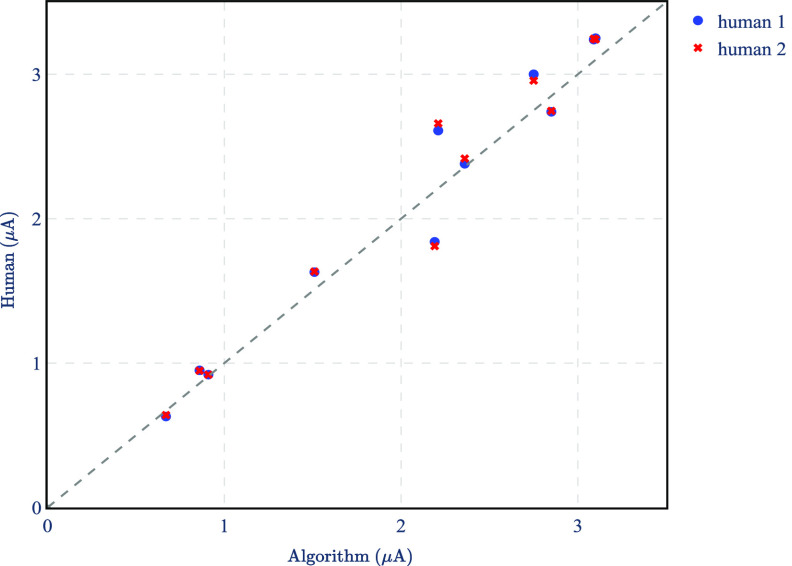
Comparison of activation thresholds between human spike sorting and the proposed method for two sets of manual analyses. The dashed line shows the identity line. Results are based on the $ex\,\,vivo$ dataset described in section 2.1.

To show an overview of the performance of the proposed spike sorting, we analyzed 10 different datasets in which there are 5 neurons stimulated by 10 different stimulating electrodes (10 neuron–electrode pairs). Figure 16 shows the activation thresholds obtained by humans and the proposed method. The stimulation thresholds estimated using the approach closely tracked those obtained with two sets of manual analyses (*R* ^2^ = 0.951 for human 1 and *R* ^2^ = 0.944 for human 2). For reference, the *R* ^2^ between human 1 and human 2 is 0.998.

## 4. Discussion

### 4.1. Spatio-temporal spike sorting

This paper proposes to exploit the different spatiotemporal characteristics of spikes and electrical stimulation artifact (figures A3 and 6(b)) to identify electrically evoked spikes in neural recordings. Our work contrasts with previous work that only exploits the different latency and amplitude of spikes and artifacts, failing to leverage the distinct spatiotemporal progression captured by high-density large-scale MEAs [23, 67]. We provided evidence that these spatiotemporal characteristics can be effectively modeled using the dynamical systems in (1) and (2), and that an input estimator can be used to retrieve the correct input sequence from MEA recordings. The activation thresholds obtained for 10 electrode–neuron pairs were similar to those obtained with human spike sorting (figure 16).

#### 4.1.1. Exploiting redundancy in high-density recordings

The proposed method can also exploit the redundancy in high-density large-scale MEA recordings to remove data from contaminated electrodes while still recovering the spatiotemporal characteristics needed to perform spike sorting. The method made it possible to ignore the stimulating electrode because it presents the largest artifact and instead exploits information available on other electrodes. This approach can increase the power and area efficiency of future hardware implementations since the analog front-end does not need to record the large artifact. Furthermore, our approach overcomes the need for modeling the artifact in the stimulating electrode, which is a non-trivial task and depends on the stimulation setup. For instance, [45] needs a different model for the stimulating electrode because it presents discontinuities and is dramatically different than the other electrodes.

Since the proposed dynamical model has more outputs than inputs, the inputs could be retrieved even in the absence of one or more electrodes. Hence, this approach could generalize to different scenarios where more than one electrode is contaminated.

### 4.2. Caveats

#### 4.2.1. Model selection

Linear models offer computational efficiency, making them appropriate for large datasets and real-time applications. Their simplicity allows for straightforward interpretation, aiding in understanding variable relationships. They also serve as a useful baseline for comparing more complex models. Additionally, linear models demonstrate robustness even when multiple models are considered, thanks to applying the *superposition principle* in underlying linear dynamical systems. However, they assume linear relationships, which may not always align with true system dynamics, potentially leading to inaccuracies with nonlinear data and limited performance [7].

In future research, exploring nonlinear models holds promise for capturing intricate dynamics that are not captured by linear analysis. However, working with nonlinear models may demand more resources and advanced analytical techniques for interpretation. Therefore, balancing increased complexity with potential gains in explanatory power will be crucial.

#### 4.2.2. Model requirements and limitations

**Figure A2.**
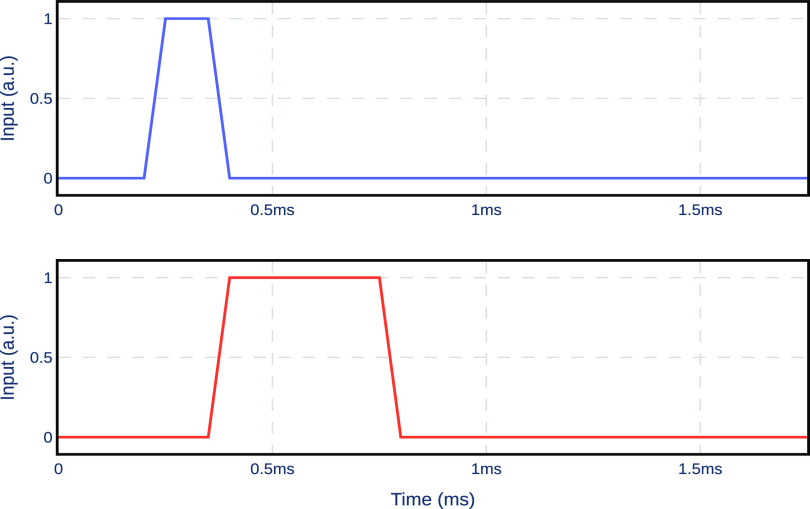
Input templates $r_{11}^t$ for neuron 11. The blue and red graphs correspond to the first and second entries of the vector $r_n^t$, respectively.

**Figure A3.**
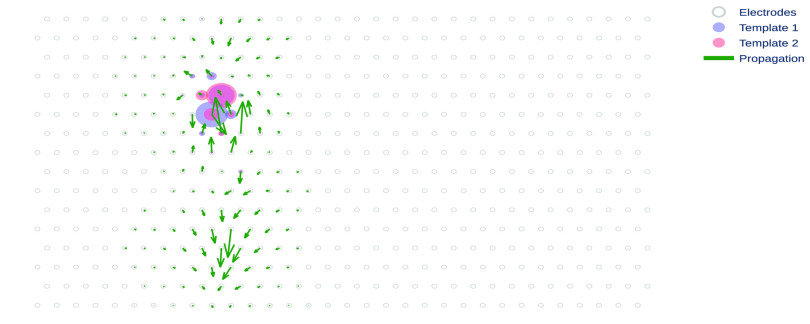
Modeled dynamics of neuron 11. The shaded red and blue colors on the electrodes show the absolute values of *B_n_*, which determines how the input template influences the dynamics of the model. The arrows show the spatial propagation of the model across electrodes, as defined by matrix *A_n_*.

The proposed approach relies on predefined input templates that are used to initiate the dynamical system and generate an output that resembles the EI of a given neuron, figure A2. Additionally, the artifact model requires input templates that fit our specific hardware system, figure B5. These input templates can be flexibly designed and adapted to match the characteristics of a specific hardware system.

We have shown that the EI models can faithfully represent the EI recordings with small errors (figure 10). However, for the neurons located on the edges of the MEA, the model is less accurate. This is likely due to incomplete information on the EI spatiotemporal propagation, which generates discontinuities in the MEA recordings.

#### 4.2.3. Computational efficiency

The low computational complexity for spike sorting is important in real-time and closed-loop applications [69]. For instance, the work proposed in [19, 45, 55, 58, 75] use computationally complex matching pursuit methods for spike sorting, where whole recordings are compared to the spiking templates of each cell. In contrast, our approach only exploits a short time window of recordings to recover input templates via the input estimator. Hence, the input estimator functions as a computationally efficient filter over the recordings similar to the Wiener filters in [51, 65]. However, further research is required to adapt this method to a real-time hardware-friendly implementation.

#### 4.2.4. Background noise

Our approach relies on the availability of EIs for each neuron obtained by the spike sorting method in [58] in the absence of stimulation. This paper does not focus on low signal-to-noise ratio (SNR) data because EIs with a peak amplitude less than $30~\mu$V are discarded. Thus, the discarded spikes are present in the background noise and may worsen the performance of the approach [63]. At the same time, the background noise is colored with spatiotemporal dependencies [16, 74], and the methods in [18, 58] can estimate its covariance. Therefore, it provides a chance to research the correlation in the background noise that may be useful in reducing the range of SNR that can be accommodated by the algorithm.

Another issue for the proposed algorithm is bundle activation, which is the result of stimulating a bundle of axons belonging to cells whose somas are outside the region covered by the MEA [22]. These spikes are also part of the background noise and may worsen the performance of the proposed method. Furthermore, if the goal is to precisely stimulate neurons at single cell and cell type resolution, then bundle activation needs to be avoided. This can be done by combining the proposed method with the method developed in [71] that detects bundle activation based on its spatiotemporal propagation characteristics.

#### 4.2.5. Artifacts

In modeling the artifact, a crucial concern is that the artifact signal is unsupervised and unknown beforehand. The stimulation data consists of the evoked neural activity, the artifact, and the background noise. Thus, we have to recover artifacts from the stimulation data to fit the artifact model. To remove the effect of noise and spontaneous neural activity, we used the average of the stimulation data from different trials similar to [45]. This works well for low-amplitude stimulation because it does not systematically lead to evoked spikes. However, as shown in figure 15(a), stimulation with high amplitudes leads to a higher probability of neuron activation. Hence, the artifact cannot be estimated by averaging for high stimulation amplitude. In literature, to model the artifact, many approaches rely on assumptions on artifact timing, lack of saturation, linearity, and decline with distance from the stimulating electrode [54, 72, 77]. Here, we simplify this task by proposing a method that does not need to model the artifact in the stimulating electrode where it is most significant and can be nonlinear.

#### 4.2.6. Hyperparameters

The models, the input estimator, and the spike detector all require hyperparameters. For the models, we experimentally fitted the hyperparameters to minimize the SNMRSE and AMRSE. For the input estimator, we tuned the hyperparameters based on the performance when estimating synthetic and real data (figures 8 and 13). For the spike detector, we relied on human annotations for adjusting hyperparameters such as detection thresholds. Hence, we require multiple human-annotated labels, which are costly and time-consuming [3, 45].

### 4.3. Towards closed-loop multi-channel stimulation

We conjecture that spatiotemporal spike sorting is suitable to enable real-time stimulation and recording at single-cell resolution in closed-loop applications. Towards this goal, future work should focus on automating the proposed method (e.g. for the hyper-parameter tuning) and designing a real-time hardware implementation.

In addition, we believe that the proposed framework can cope with multi-channel stimulation scenarios. This could have a large impact in basic neuroscience and clinical applications since multi-channel stimulation enables an effective strategy to spatially control the spiking of multiple neurons [34, 39]. To address the multi-channel stimulation scenario, the proposed method could factor in multiple artifact models in the aggregate model and retrieve the input templates of EIs in the presence of different artifacts simultaneously.

## Figures and Tables

**Figure A1.**
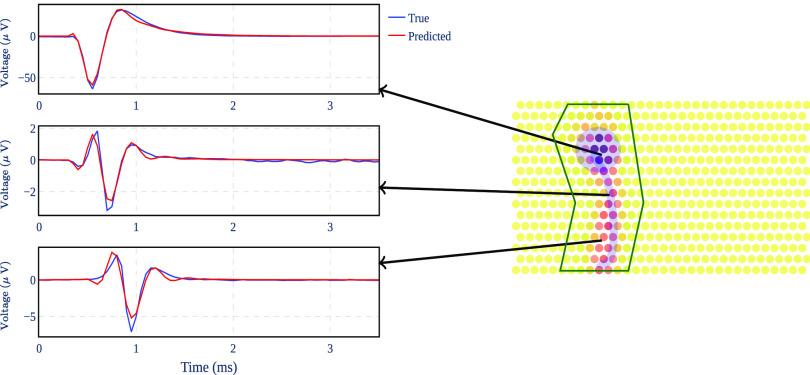
Recorded and predicted EI for neuron 11. The heatmap shows the footprint of the neuron on the MEA; the color encodes the root mean square (RMS) of each electrode’s recordings when a spike from the putative neuron is present. The green region encloses the electrodes selected as states in the model.

**Figure B4.**
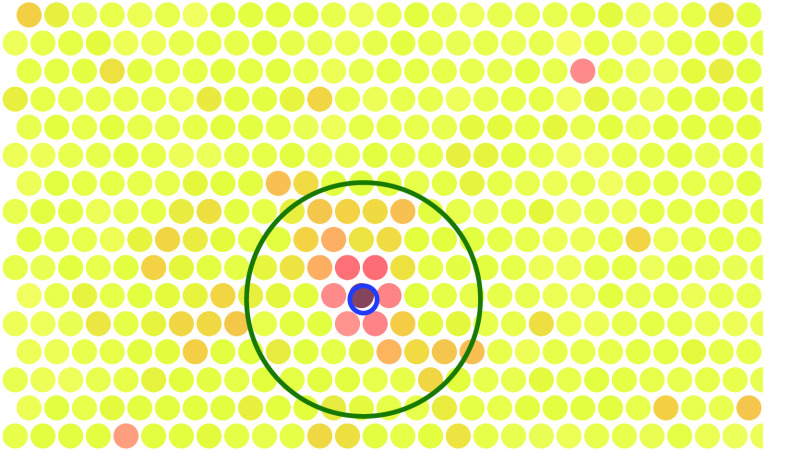
The heat-map of the artifact on the MEA. The blue circle indicates the stimulating electrode, and the green circle encloses the electrodes considered as part of the state.

**Figure B6.**
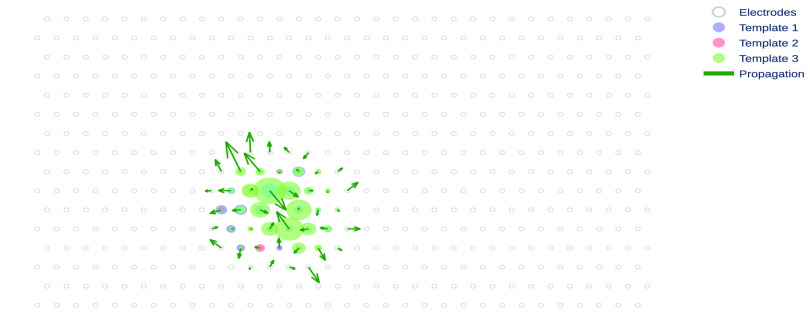
Dynamics of the artifact on MEAs. The arrows show the direction in which each electrode’s measured activity is propagated according to *A*_*a*_ *x*. The shaded colors on the electrodes show the absolute values of *B_a_*, which determines how three input templates of the EI model change the activity of the electrodes.

## Data Availability

All data that support the findings of this study are included within the article (and any supplementary files).

## Software routines

To maintain transparency and facilitate reproducibility in line with the FAIR guiding principles, we provide full access to the software routines used to generate the empirical results presented in this paper.

The code repository, hosted on GitHub, can be found at the following link: https://github.com/mohammadshokriacct/electrical_spike_sorting. Our routines are written in Python, leveraging the robust functionalities of TensorFlow and Keras libraries, which are well-suited for complex computational tasks such as those performed in our study.

## Appendix A Electrical image model

The electrical image (EI) of a neuron is the average time-series recording across all electrodes when the neuron is firing a spike. Here, we record from *E* = 512 electrodes for *T* = 71 time samples corresponding to 3.55 ms (sampling rate equal to 20 kS s^−1^) for each EI, and we collect EIs from *N* = 25 distinct neurons. To represent the EI for neuron *n*, we use the following dynamical system:

\begin{align*} \begin{aligned} x_n^{t+1} &amp; = A_n x_n^t + B_n u_n^t, \\ y_n^t &amp; = C_n x_n^t. \end{aligned} \end{align*}

### A.1. Input, states and output

Here, we define the EI for neuron *n* as the EI model output $\{y_n^t\}_{t = 1}^{T}\in \mathbb{R}^E$. The $E_n\lt E$ electrodes that exhibit a maximum absolute value larger than a pre-defined state-threshold $\theta_n^S \gt0$ are selected as the EI model state variables $x_n^t\in \mathbb{R}^{E_n}$ (green region in figure A1). The input to the model is $u_n^t\in\mathbb{R}^2$. When the input $u_n^t$ is identical to the input template $r_n^t \in \mathbb{R}^2$ (figure A2), the output is the EI of neuron *n*. The input template is defined by four parameters pertaining to the starting and ending points of the two pulses. These parameters are tuned to minimize the mean square error between the model output and the original EI.

### A.2. Matrices *A_n_* and *B_n_*

*A_n_* determines how each state (i.e. selected electrode) influences the future states, including itself. Here, we assume that a given electrode is unlikely to influence further away electrodes due to the slow spatiotemporal progression of the neural signal across the MEA [1, 5, 29, 53, 72]. Hence, if *d*_*ij*_ is the distance between electrode *i* and *j*, the element of *A_n_* on the row *i* and column *j* is set to zero, providing that $d_{ij}\gt\theta_n^A$ (predefined threshold).

*B_n_* determines how the input influences the future states. The input is assumed to influence only the electrodes close to the soma of the neuron. The electrode $i^\star$ that records the maximum amplitude in the EI is considered the base electrode. If $d_{i^\star j}$ is the distance between the base electrode and the electrode *j*, the elements of *B_n_* on the column *j* are set to zero, providing that $d_{i^\star j}\gt\theta_n^B$ (predefined threshold).

To find *A_n_* and *B_n_*, we perform system identification using the EI data as states and the input templates as inputs [30, 43, 52, 76]. To obtain the parameters, we minimize the error of the dynamical equation for all data points. Notice that this minimization is performed subject to the structure of the matrices, where some elements of *A_n_* and *B_n_* are set to zero based on the thresholds $\theta_n^A$ and $\theta_n^B$. Figure A3 illustrates the effect of *A_n_* and *B_n_*.

### A.3. Matrix *C_n_*

Here, the output corresponds to the state for the selected electrodes and is approximated to zero for the other electrodes. The matrix *C_n_* is an identity matrix for the columns corresponding to the selected electrodes and zero for the columns corresponding to the non-selected electrodes.

The procedure for the EI model is described in algorithm 1. First, we determine the state variables of the dynamical system. Then, we determine the structure of the matrices of the model. Finally, based on the structure of the model, we fit the matrices using the EI data for the states.

**Table:**

**Algorithm 1.** System identification for modeling of EI *n*.
**Algorithm’s inputs**
EI data of neuron *n* $\{y_n^t\}_{t = 1}^{T}\in \mathbb{R}^E$
Input template of neuron *n* described by $\{r_n^t\}_{t = 1}^{T}\in \mathbb{R}^2$
*d*_*ij*_ distance between electrode *i* and electrode *j*
*A* matrix threshold $\theta_n^A$
*B* matrix threshold $\theta_n^B$
State threshold $\theta_n^S$
**State Selection**
Calculate $p_{n,i} = \max_{t}||y_{n,i}^t||$ for electrode *i* where $y_{n,i}^t$ is the element *i* of $y_n^t$
State set to contain the electrodes ${\cal E}^S_n = \{i|p_{n,i}\unicode{x2A7D}\theta_n^S\}$ to be considered as states
Number of states given by $E_n = |{\cal E}^S_n|$
State vector $x_n^t = [{\,}y_i^t] \in \mathbb{R}^{E_n}$, where $i\in {\cal E}^S_n$
**Matrix Structures**
*A* structure: $A\in \mathbb{R}^{E_n\times E_n}$ where $a_{ij} = 0$ for electrodes $i\in {\cal E}^S_n$ and $j\in {\cal E}^S_n$ such that $d_{ij}\gt\theta_n^A$
Base electrode: $i^* = \textbf{argmax}_{i\in {\cal E}^S_n}p_{n,i}$
*B* structure: $B\in \mathbb{R}^{E_n\times 2}$ where $b_{ij} = 0$ for electrodes $i\in {\cal E}^S_n$ and $j = 1,2$ such that $d_{i i^\star}\gt\theta_n^B$
**System Identification**
$(A_n, B_n) = \textbf{argmin}_{A, B} \sum\limits_{t = 1}^{T-1} ||x_n^{t+1} - (A x_n^t + B r_n^t)||^2$ subject to the matrices’ structures
Set $C_n = I_{E}$, and remove the columns $i \notin {\cal E}^S_n$ of *C_n_* such that $C_n\in \mathbb{R}^{E \times E_n}$
**Return** *A_n_*, *B_n_*, *C_n_*

## Appendix B Artifact modeling

Stimulation data is a time-series $\{y_k^{t,q}\}_{t = 1}^{T}\in \mathbb{R}^E$ at the stimulation amplitude $q\in {\cal Q}$ for trials $k = 1,\dots,K$, where *K* = 20. ${\cal Q}$ represents the set of 39 different stimulation amplitudes from $0.11~\mu$A to $3.71~\mu$A. To represent the stimulation artifact, we use the following dynamical system:

\begin{align*} \begin{aligned} x_a^{t+1} &amp; = A_a x_a^t + B_a u_a^t, \\ y_a^t &amp; = C_a x_a^t. \end{aligned} \end{align*}

### B.1. Input, states, and output

To model the artifact, we use the average time-series data across different trials as the output of the system $\{y^{t,q}\}_{t = 1}^{T} = \frac{1}{K}\sum_{k = 1}^{K}y_k^{t,q}$. The average signal is used to mitigate the effect of spontaneous, ubiquitous spikes on the MEA. Here, we select the electrodes close to the stimulation electrode as the states of the model (see the green region in figure B4). Specifically, the states are the electrodes at a distance less than the predefined threshold $\theta_a^S$ that belong to the set ${\cal E}^S_a$ with *E_a_* members. Hence, $x_a^t\in \mathbb{R}^{E_a}$ stands for the states of the artifact model at time *t*. Notably, the stimulating electrode is discarded from the states because of its nonlinear behavior at different amplitudes. The input to the model is $u_a^t\in\mathbb{R}^3$. When the input $u_a^t$ is identical to the input template $r_a^t \in \mathbb{R}^3$ (figure B5), the output is the artifact. The input template is defined by six parameters pertaining to the starting and ending points of the three pulses. These parameters are tuned to minimize the mean square error between the model output and the artifact for the lowest amplitude. For the other amplitudes, the input templates are scaled by the stimulation amplitude.

### B.2. Matrices *A_a_*, *B_a_* and *C_a_*

The matrices for the artifact model are obtained similarly to the ones for the EI model. Distance thresholds $\theta_a^A$ and $\theta_a^B$ are set to define the effect of the current states and inputs on the future states (figure B6). Matrices *A_a_* and *B_a_* are designed to minimize the error of the output for different stimulation amplitudes using system identification.

The procedure for the artifact model is detailed in algorithm 2. First, we determine the electrodes to be used as states. Then, we determine the structure of the matrices *A_a_* and *B_a_*. In the end, the matrices of the model are fitted with the state data.

**Table:**

**Algorithm 2.** System identification for artifact modeling.
**Algorithm’s inputs**
The set of stimuli amplitudes ${\cal Q}$
Average of all trials for stimulated data $\{y^{t,q}\}_{t = 1}^{T}\in \mathbb{R}^E$ at all stimuli amplitude $q\in {\cal Q}$
Artifact input template $\{r_a^t\}_{t = 1}^{T}\in \mathbb{R}^3$
Stimulating electrode $i^\star$
*d*_*ij*_ distance between electrode *i* and electrode *j*
*A* matrix threshold $\theta_a^A$
*B* matrix threshold $\theta_a^B$
State threshold $\theta_a^S$
**State Selection**
State set to contain the electrodes that satisfy ${\cal E}^S_a = \{i|d_{i i^\star}\unicode{x2A7D}\theta_a^S\}$
Number of states given by $E_a = |{\cal E}^S_a|$
State vector $x_a^{t,q} = [{\,}y_i^{t,q}] \in \mathbb{R}^{E_a}$ at the stimuli amplitude *q*, where $i\in {\cal E}^S_a$
**Matrix Structures**
*A* structure: $A\in \mathbb{R}^{E_a\times E_a}$ where $a_{ij} = 0$ for electrodes $i\in {\cal E}^S_a$ and $j\in {\cal E}^S_a$ such that $d_{ij}\gt\theta_a^A$
*B* structure: $B\in \mathbb{R}^{E_a\times 3}$ where $b_{ij} = 0$ for $j = 1,2,3$ and electrodes $i\in {\cal E}^S_a$ such that $d_{i i^\star}\gt\theta_a^B$
**System Identification**
$u_a^{t,q} = q r_a^t$ at the stimuli amplitude $q\in {\cal Q}$ $(A_a, B_a) = \textbf{argmin}_{A, B} \sum\limits_{q\in{\cal Q}}\sum\limits_{t = 1}^{T-1} ||x_a^{t+1,q} -$ $(A x_a^{t,q} + B u_a^{t,q})||^2$ subject to the matrices’ structures
Set $C_a = I_{E}$, and remove the columns $i \notin {\cal E}^S_a$ of *C_a_* such that $C_a\in \mathbb{R}^{E \times E_a}$
**Return** *A_a_*, *B_a_*, *C_a_*

## Appendix C Aggregate model

The aggregate model describes the propagation of the voltage across electrodes in the presence of both stimulation artifact and neurons firing. This model is a linear combination of the EI model for all neurons, the artifact model and the measurement noise (superposition assumption in MEA recordings [58]). The model output is assumed to be the sum of the output of all EI models, the artifact model and noise (*w*), i.e. $y^t = \sum_{n = 1}^{N}y_n^t + y_a^t + w^t$ at time *t*. We can reformulate the final output as follows:

\begin{align*} \begin{aligned} y^t&amp; = \sum_{n = 1}^{N}C_n x_n^t + C_a x_a^t + w^t\\ &amp; = \left[\begin{array}{cccc} C_1 &amp; \cdots &amp; C_N &amp; C_a \end{array}\right]{\tilde x}^t + w^t = {\tilde C} {\tilde x}^t + w^t , \end{aligned} \end{align*}

where ${\tilde C} = \left[\begin{array}{cccc} C_1 &amp; \cdots &amp; C_N &amp; C_a \end{array}\right]$ and ${\tilde x} = \left[ x_1^\top,\dots, x_N^\top, x_a^\top \right]^\top$ is the aggregate state. To describe how the aggregate state evolves, we aggregate the dynamics of each model into the following model:

\begin{align*} \begin{aligned} {\tilde x}^{t+1} &amp; = {\tilde A} {\tilde x}^t + {\tilde B} {\tilde u}^t + {\tilde B}^{\prime} u_a^t, \end{aligned} \end{align*}

where ${\tilde u} = \left[ u_1^\top,\dots, u_N^\top \right]^\top$ is the aggregate input for all neurons, and the matrices are calculated as

\begin{align*} \begin{aligned} {\tilde A} &amp; = \left[\begin{array}{cccc} A_1 &amp; &amp; &amp; 0 \\ &amp; \ddots &amp; &amp; \\ &amp; &amp; A_N &amp; \\ 0 &amp; &amp; &amp; A_a \\ \end{array}\right], {\tilde B} = \left[\begin{array}{ccc} B_1 &amp; &amp; 0 \\ &amp; \ddots &amp; \\ 0 &amp; &amp; B_N \\ 0 &amp; \dots &amp; 0 \\ \end{array}\right], \text{ and } \\ {\tilde B}^{\prime} &amp; = \left[\begin{array}{c} 0 \\ \vdots \\ 0 \\ B_a \\ \end{array}\right]. \end{aligned} \end{align*}

## Appendix D Input estimator

The input estimator aims to estimate the input sequence and the states that likely generated the electrode recordings using the proposed aggregate model. The estimated inputs are from different models and allow us to differentiate which models (i.e. neurons) contribute to the measurement. The structure of the input estimator is depicted in figure 7(b). Here, the input estimator leverages the aggregate model to estimate the input sequence and the states of the system using a window of size *L* of the output data (i.e. electrode recordings).

Based on the aggregate model, we can define the input-to-output relationship as follows:

\begin{align*} \begin{aligned} y^{t:t+L} = O_L {\tilde x}^t + J_L {\tilde u}^{t:t+L} + J_L^{\prime} u_a^{t:t+L} + w^{t:t+L}, \end{aligned} \end{align*}

where $y^{t:t+L} = \left[ (y^{t})^\top,\dots,(y^{t+L})^\top \right]^\top$ is the output data, ${\tilde u}^{t:t+L} = \left[ ({\tilde u}^{t})^\top,\dots,({\tilde u}^{t+L})^\top \right]^\top$ is the aggregate inputs of the EI models, $u_a^{t:t+L} = \left[ (u_a^{t})^\top,\dots,(u_a^{t+L})^\top \right]^\top$ is the input of the artifact model, and $w^{t:t+L} = \left[ (w^{t})^\top,\dots,(w^{t+L})^\top \right]^\top$ is the measurement noise within a forward window *L* [41]. The matrices in (A6) are computed as

\begin{align*} \begin{aligned} O_L &amp; = \left[\!\!\begin{array}{@{}c} {\tilde C} \\ {\tilde C}{\tilde A} \\ \vdots \\ {\tilde C}{\tilde A}^L \end{array}\!\!\right], \,\,\,\, J_L = \left[\begin{array}{@{}ccccc} 0 &amp; &amp; &amp; &amp;0 \\ {\tilde C}{\tilde B} &amp; 0 &amp; &amp; &amp; \\ {\tilde C}{\tilde A}{\tilde B}&amp; {\tilde C}{\tilde B} &amp; 0 &amp; &amp; \\ \vdots &amp; \ddots &amp; \ddots &amp; \ddots &amp; \\ \\ {\tilde C}{\tilde A}^{L-1} {\tilde B} &amp; \dots &amp; {\tilde C}{\tilde A}{\tilde B} &amp; {\tilde C}{\tilde B} &amp; 0 \\ \end{array}\right], \,\,\,\, \text{and} \\ J_L^{\prime} &amp; = \left[\begin{array}{@{}ccccc} 0 &amp; &amp; &amp; &amp;0 \\ {\tilde C}{\tilde B}^{\prime} &amp; 0 &amp; &amp; &amp; \\ {\tilde C}{\tilde A}{\tilde B}^{\prime}&amp; {\tilde C}{\tilde B}^{\prime} &amp; 0 &amp; &amp; \\ \vdots &amp; \ddots &amp; \ddots &amp; \ddots &amp; \\ \\ {\tilde C}{\tilde A}^{L-1} {\tilde B}^{\prime} &amp; \dots &amp; {\tilde C}{\tilde A}{\tilde B}^{\prime} &amp; {\tilde C}{\tilde B}^{\prime} &amp; 0 \\ \end{array}\right]. \end{aligned} \end{align*}

If data from $y^{t:t+L}$, ${\tilde x}^t$, and $u_a^{t:t+L}$ is available, we can estimate ${\tilde u}^{t:t+L}$ from (A6) and obtain the state estimation for the next time based on the aggregate model. In [9], this problem is addressed by using an unknown input observer (UIO) that aims to estimate the states when the input signal is unknown. The UIO estimates the next state as follows:

\begin{align*} \begin{aligned} z^{t+1} = E^t {\hat x}^t + F^t y^{t:t+L} + F_a^t u_a^{t:t+L}, \end{aligned} \end{align*}

where $z^{t+1}$ is the unconstrained estimation of the states at time *t* + 1, and *E^t^*, *F^t^*, and $F_a^t$ are the filters on the available data for the last estimation, the output sequence, and the artifact input (the derivation of these filters is explained later). Note that the inputs to the artifact model are available, and the inputs to the EI models are the unknown inputs in our setting. Since each EI input is in the interval $[0,1]$, the unconstrained estimation of the states $z^{t+1}$ from (A8) may be wrong. Therefore, we obtain the estimated input ${\hat u}^t$ from the following optimization:

\begin{align*} \begin{aligned} {\hat u}^t = \textbf{argmin}_{u \in \left[0,1\right]^{2N}} ||z^{t+1} - {\tilde A} {\hat x}^t - {\tilde B} u - {\tilde B}^{\prime} u_a^t||^2, \end{aligned} \end{align*}

where *N* is the number of neurons. The optimization (A9) enforces that the estimated input is the nearest input in the interval $[0,1]$ that leads to the unconstrained estimated state. From ${\hat u}^t$, the estimated state at time *t* + 1 can be calculated as:

\begin{align*} \begin{aligned} {\hat x}^{t+1} = {\tilde A} {\hat x}^t + {\tilde B} {\hat u}^t + {\tilde B}^{\prime} u_a^t. \end{aligned} \end{align*}

Algorithm 3 shows the procedure of state and input estimation from the output data. In the algorithm, we considered that we have the matrices *E^t^*, *F^t^*, and $F_a^t$ from the UIO design (computed by the function ${\cal G}({\cal I}, t, t + L)$). ${\cal I}$ stands for the sets of unavailable measurements.

**Table:**

**Algorithm 3.** Procedure of input estimation.
**Algorithm’s inputs**
Output data $\{y^t\}_{t = 1}^{T}\in \mathbb{R}^E$
Aggregate model parameters ${\tilde A}$, ${\tilde B}$, and ${\tilde B}^{\prime}$
Artifact input $\{u_a^t\}_{t = 1}^{T}\in \mathbb{R}^E$
Corrupted electrode indices ${\cal I}$
Window length *L*
Initial state estimation ${\hat x}^0$
**Input Estimation Procedure:**
*t* = 0
**While** $t \lt = T - L$:
$E^t, F^t, F_a^t = {\cal G}({\cal I}, t, t + L)$
$y^{t:t+L} = \left[ (y^{t})^\top,\dots,(y^{t+L})^\top \right]^\top$
$u_a^{t:t+L} = \left[ (u_a^{t})^\top,\dots,(u_a^{t+L})^\top \right]^\top$
$z^{t+1} = E^t {\tilde x}^t + F^t y^{t:t+L} + F_a^t u_a^{t:t+L}$
${\hat u}^t = \textbf{argmin}_{u \in [0,1]^{2N}} ||z^{t+1} - {\tilde A} {\hat x}^t - {\tilde B} u - {\tilde B}^{\prime} u_a^t||^2$
${\hat x}^{t+1} = {\tilde A} {\hat x}^t + {\tilde B} {\hat u}^t + {\tilde B}^{\prime} u_a^t$
$t = t + 1$
**End While**

By applying (A6) into (A8) and subtracting (A4), we have

\begin{align*} \begin{aligned} e_z^{t+1} &amp; = E^t e_x^t + \left(-E^t+{\tilde A}- F^t O_L\right){\tilde x}^t \\ &amp; \quad + \left(\left[\begin{array}{cc} {\tilde B} &amp; 0 \end{array}\right]- F^t J_L\right) {\tilde u}^{t:t+L} \\ &amp; \quad + \left(\left[\begin{array}{cc} {\tilde B}^{\prime} &amp; 0 \end{array}\right]- F^t J_L^{\prime} - F_a^t\right) u_a^{t:t+L} - F^t w^{t:t+L}, \end{aligned} \end{align*}

where $e_x^t = {\tilde x}^t-{\hat x}^t$ is the estimation error, and $e_z^{t+1} = {\tilde x}^{t+1}-z^{t+1}$ is the unconstrained estimation error. In order to force the error to go to zero regardless of the state and input, the following equations should hold:

\begin{align*} \begin{aligned} E^t &amp; = {\tilde A}- F^t O_L,\\ F^t J_L &amp; = \left[\begin{array}{cc} {\tilde B} &amp; 0 \end{array}\right],\text{and } \\ F_a^t &amp; = \left[\begin{array}{cc} {\tilde B}^{\prime} &amp; 0 \end{array}\right] - F^t J_L^{\prime}. \end{aligned} \end{align*}

If (A12) holds, we conclude that $\mathbb{E}\{e_z^{t+1}\} = E^t \mathbb{E}\{e_x^t\}$ for estimation errors according to (A11). To obtain the matrices that satisfy (A12), we follow the procedure of [9] for the fully-observed situation where the data from all electrodes is available. In this scenario, the matrices *E^t^*, *F^t^*, and $F_a^t$ can be calculated regardless of time because the other matrices in the equation (A12) are time-invariant. In this work, we first calculate the parameters of the UIO for a fully-observed situation where the matrices are time-independent ($E^*$, $F^*$ and $F_a^*$ denote the solution), and then, we extend it to a partially-observed situation where some electrodes are excluded.

To solve (A12), the authors in [9] propose a method where the matrices *M*, *S*_1_, and *S*_2_ are calculated such that

\begin{align*} \begin{aligned} M J_L &amp; = \left[\begin{array}{cc} 0 &amp; 0 \\ I_{2N} &amp; 0 \end{array}\right], \text{ and }\\ \left[\begin{array}{c} S_1 \\ S_2 \end{array}\right] &amp; = M O_L, \end{aligned} \end{align*}

where $I_{2N}$ is the identity matrix with 2*N* dimension. Hence, the solution of (A12) can be calculated as

\begin{align*} \begin{aligned} E\left(X\right) &amp; = {\tilde A} - {\tilde B} S_2 - X S_1,\\ F\left(X\right) &amp; = \left[\begin{array}{cc} X &amp; {\tilde B} \end{array}\right] M, \text{ and }\\ F_a\left(X\right) &amp; = \left[\begin{array}{cc} {\tilde B}^{\prime} &amp; 0 \end{array}\right] - F\left(X\right) J_L^{\prime}, \end{aligned} \end{align*}

where *X* is the degree of freedom for the solution. To find *X*, we solve the following optimization problem:

\begin{align*} \begin{aligned} X^* &amp; = \textbf{argmin}_{X} ||E\left(X\right)||^2_F \\ &amp; \quad + \alpha \sum_{t = 1}^{T-L} \sum_{n = 1}^{N} ||Y_n F\left(X\right) y_n^{t:t+L}||^2_F, \end{aligned} \end{align*}

where *Y_n_* is a diagonal matrix where its diagonal element is equal to 1, except for the elements regarding the states of neuron *n* from ${\tilde A}$ pattern, and $||.||_F$ is the Frobenius norm of matrices. Also, $y_n^t$ is the EI data of neuron *n*, and *α* is a positive coefficient. The term $||E(X)||^2_F$ of the optimization leads the estimator to converge faster, and the term $\sum_{t = 1}^{T-L} \sum_{n = 1}^{N} ||Y_n F(X) y_n^{t:t+L}||^2_F$ makes the estimator be robust against the spontaneous firing of other neurons. Therefore, the solution of (A12) is calculated by $E^* = E(X^*)$, $F^* = E(X^*)$, and $F_a^* = F_a(X^*)$. Algorithm 4 shows the design of the fully-observed UIO. To design the UIO, we need to define the parameter *L* such that it satisfies the following constraint [9]:

\begin{align*} \begin{aligned} {\mathrm {rank}}\left(J_L\right) - {\mathrm {rank}}\left(J_{L-1}\right) = 2N. \end{aligned} \end{align*}

The constraint (A16) is an essential condition for obtaining the matrices *M*, *S*_1_, and *S*_2_ in algorithm 4. In this work, we considered *L* = 10, and we evaluated the satisfaction of (A16) during the algorithm.

**Table:**

**Algorithm 4.** Fully-observed UIO design.
**Algorithm’s inputs**
Output data $\{y^t\}_{t = 1}^{T}\in \mathbb{R}^E$
Aggregate model parameters ${\tilde A}$, ${\tilde B}$, and ${\tilde B}^{\prime}$
Window length *L*
Regularization coefficient *α*
**If** *L* does not satisfy (A16):
Exit
**Algebraic procedure of [9]:**
Calculate the matrices *M*, *S*_1_, and *S*_2_ to satisfy (A13)
$X^* = \textbf{argmin}_{X} ||E(X)||^2_F + \alpha \sum_{t = 1}^{T-L} \sum_{n = 1}^{N}$ $||Y_n F(X) y_n^{t:t+L}||^2_F$
$E^* = {\tilde A} - {\tilde B} S_2 - X^* S_1$
$F^* = \left[\begin{array}{cc} X^* &amp; {\tilde B} \end{array}\right] M$
$F_a^* = \left[\begin{array}{cc} {\tilde B}^{\prime} &amp; 0 \end{array}\right] - F^* J_L^{\prime}$
**Return** $E^*$, $F^*$, $F_a^*$

Algorithm 4 designs the UIO for a fully-observed situation where the UIO uses the data of all electrodes. For a partially-observed situation, we suppose that some electrodes are not available (e.g. the stimulating electrode), and indicate this with the set ${\cal I}$. The pair $(e,t)\in {\cal I}$ means that the measurement from electrode *e* is not available at time *t*. Algorithm 5 shows the design for a partially-observed UIO. Whereas in the fully-observed solution, $F^*$ satisfies (A12), in the partially-observed case, we obtain the closest matrix *F* to $F^*$ with the available electrodes.

**Table:**

**Algorithm 5.** Partially-observed UIO.
**Algorithm’s inputs**
Window length *L*
Aggregate model parameters ${\tilde A}$
Input-output parameters *J_L_*, $J_L^{\prime}$, and *O_L_*
Full information parameters of UIO $F^*$
**Function** ${\cal G}({\cal I}, t_{\mathrm {start}}, t_{\mathrm {end}})$:
$J = J_L$, $J^{\prime} = J_L^{\prime}$, and $O = O_L$
Remove the row $L(t-t_{\mathrm {start}})+e$ of *J*, *J*ʹ, and *O* suchthat $(e,t) \in {\cal I}$ and $t_{\mathrm {start}}\unicode{x2A7D} t \unicode{x2A7D} t_{\mathrm {end}}$
$F_{\mathrm {new}} = \textbf{argmin}_{F} ||J F - J_L F^*||^2$
$F_{\mathrm {new}}^{\prime} = \left[\begin{array}{cc} B^{\prime} &amp; 0 \end{array}\right] - F_{\mathrm {new}} J^{\prime}$
$E_{\mathrm {new}} = {\tilde A} - F_{\mathrm {new}} O$
**Return** $E_{\mathrm {new}}$, $F_{\mathrm {new}}$, $F_{\mathrm {new}}^{\prime}$

## Appendix E Spike normalized root mean square error

We use the spike normalized root mean square error (SNRMSE) to assess the performance of the EI models. We apply normalization to compare neurons with different spike amplitudes. For neuron *n*, consider $y_n^t$ and $z_n^t$ for $t = 1,\dots,T$, to be the real and predicted value of EI *n*, respectively. SNRMSE for EI *n* is calculated as follows:

\begin{align*} {\mathrm {SNRMSE}}_n = \frac{\sqrt{\frac{1}{T}\sum_{t = 1}^{T}||y_n^t - z_n^t||^2}}{\sqrt{\frac{1}{T}\sum_{t = 1}^{T}||y_n^t||^2}}. \end{align*}

## Appendix F Artifact normalized root mean square error

We use the artifact normalized root mean square error (ANRMSE) to assess the performance of the artifact model. Notice that the electrode measurements after stimulation include the stimulation artifact, spontaneous and elicited spikes, and noise. Hence, to evaluate the artifact model, we should remove the effect of spikes and noise from the measurement. Especially, spikes have a major effect during high-amplitude stimulation trials where the probability of eliciting a spike increases. Consider the electrode measurement *y^t^* and let ${\hat y}_n^t$ be the estimated output of the activated neurons (by the input estimator). The output $y_0^t = y^t - \sum_{n = 1}^{N} {\hat y}_n^t$ describes the measurement without the effect of neuron(s) estimated to be elicited. Finally, let us define $z_a^t$ as the prediction of the artifact model. Therefore, ANRMSE is calculated as follows:

\begin{align*} {\mathrm {ANRMSE}} = \frac{\sqrt{\frac{1}{T}\sum_{t = 1}^{T}||y_0^t - z_a^t||^2}}{\sqrt{\frac{1}{T}\sum_{t = 1}^{T}||y_0^t||^2}}. \end{align*}

## Appendix G Spike sorting

To detect spikes from the estimated inputs, we propose some indices that show the similarity of the estimated inputs and their templates. Since this similarity may happen at any time, we compare the estimated input signal and input templates for *T* sample interval (for our case, *K* = 15). To do so, we used the squared error (SE) as a similarity function as follows:

\begin{align*} {SE}_n^t = \sum_{k = 0}^{K}||u_n^{t+k} - r_n^k||^2, \end{align*}

where ${SE}_n^t$ is SE for neuron *n* at time *t*. The smaller ${SE}_n^t$, the higher the similarity between the estimated inputs and the templates. Therefore, the threshold *θ*_*SE*_ is considered for the spike detection such that the satisfaction of ${SE}_n^t \lt \theta_{SE}$ leads to spike presence in the estimated input.
